# A set of conformationally well-defined L/D-peptide epitopes provides a serological bar code for autoantibody subtypes

**DOI:** 10.1371/journal.pone.0201735

**Published:** 2018-08-03

**Authors:** Andreas Schrimpf, Dörte Brödje, Petra Pfefferle, Armin Geyer

**Affiliations:** 1 Department of Chemistry, Philipps-Universität Marburg, Marburg, Germany; 2 Department of Medicine, Philipps-Universität Marburg, Marburg, Germany; 3 Comprehensive Biomaterial Bank Marburg (CBBMR), Marburg, Germany; Duke University School of Medicine, UNITED STATES

## Abstract

Which conformational parameters lead to an antibody-affine peptide antigen? And in how many different conformations can we actually present the respective conformational epitope? To provide answers from a chemical point of view, we direct the bending and tethering of peptide backbones by the utilisation of a hydrophobic cluster, disulfides, and d-amino acids. Each mutation is employed pairwise on directly opposite sides of a β-hairpin. In combination, these synthetic modules guide the formation of complementary β-sheet-like structures, whereby the oppositely configured (l/d-)bi-disulfide pairs form with high regioselectivity. The conformational properties of the peptides are assessed by NMR spectroscopy and correlated with their antibody affinity in ELISA. From a pool of thus designed peptide antigens with distinctive complementary affinities against known rheumatoid arthritis (RA) autoantibodies, we select a set of epitopes for an immunoassay with sera of RA patients. We want to put emphasis on the idea, that the different conformational properties of the chosen antigens, containing the same epitope sequence, are mirrored in the distribution of autoantibody subtypes (or of the antibody polyclonality, respectively). Such directly comparable information can only be delivered by a set of peptides, rather than a single one. The hairpin-restriction technology of l/d-configured bi-disulfide amino acid pairs is not limited to RA but applicable to other shape-persistent hairpin motifs which are supposed to identify subgroups of protein receptors.

## Introduction

**Fig 1:**
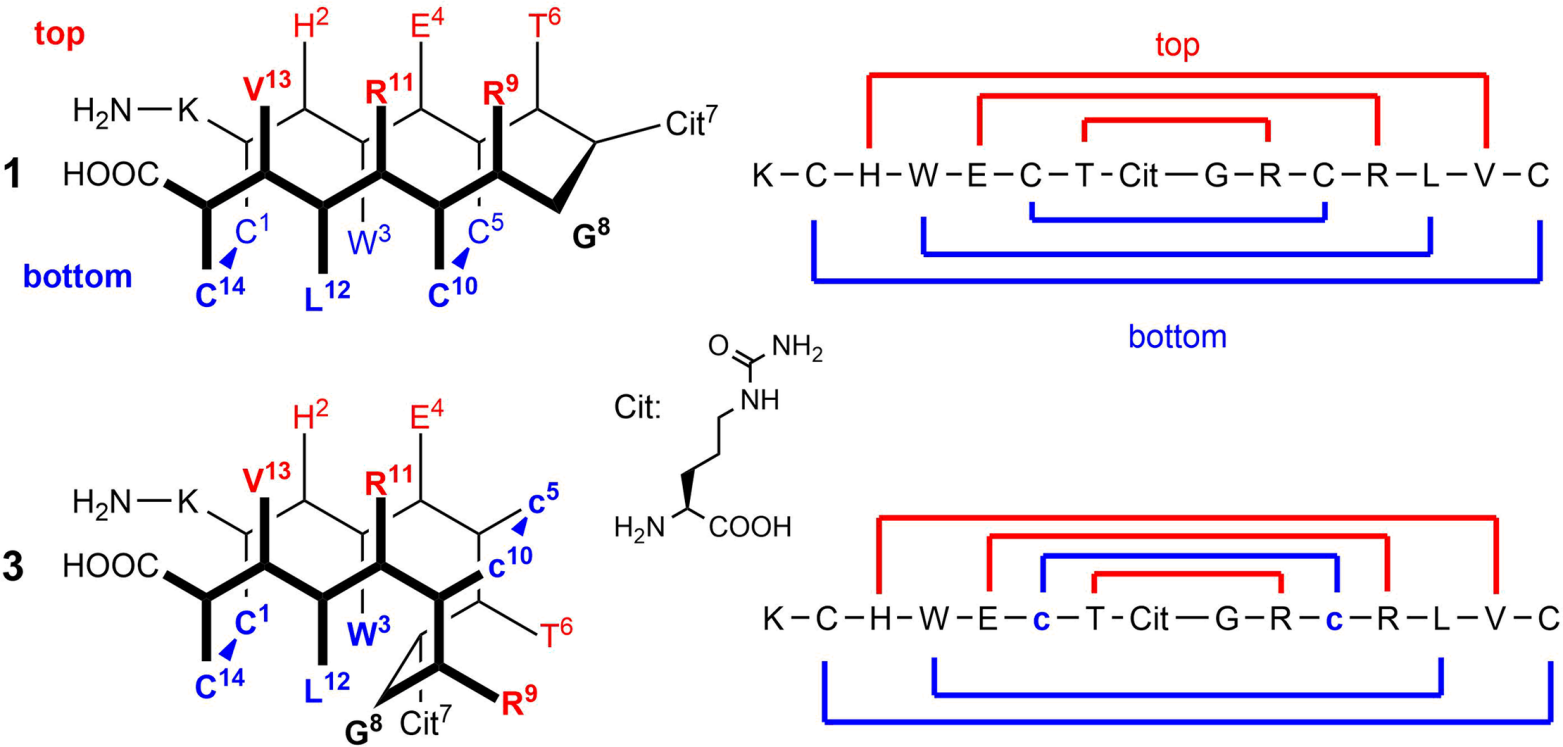
Simplified zigzag representation of utilised β-hairpin peptides. The pictograms show the relative orientation of the side chains on the upper (red) and lower side (blue) of the secondary structure in the all-l bi-disulfide **1** (Cit: l-citrulline). A pair of d-configurated amino acids flips the side chains to the other side as shown for peptide **3**.

**Fig 2:**
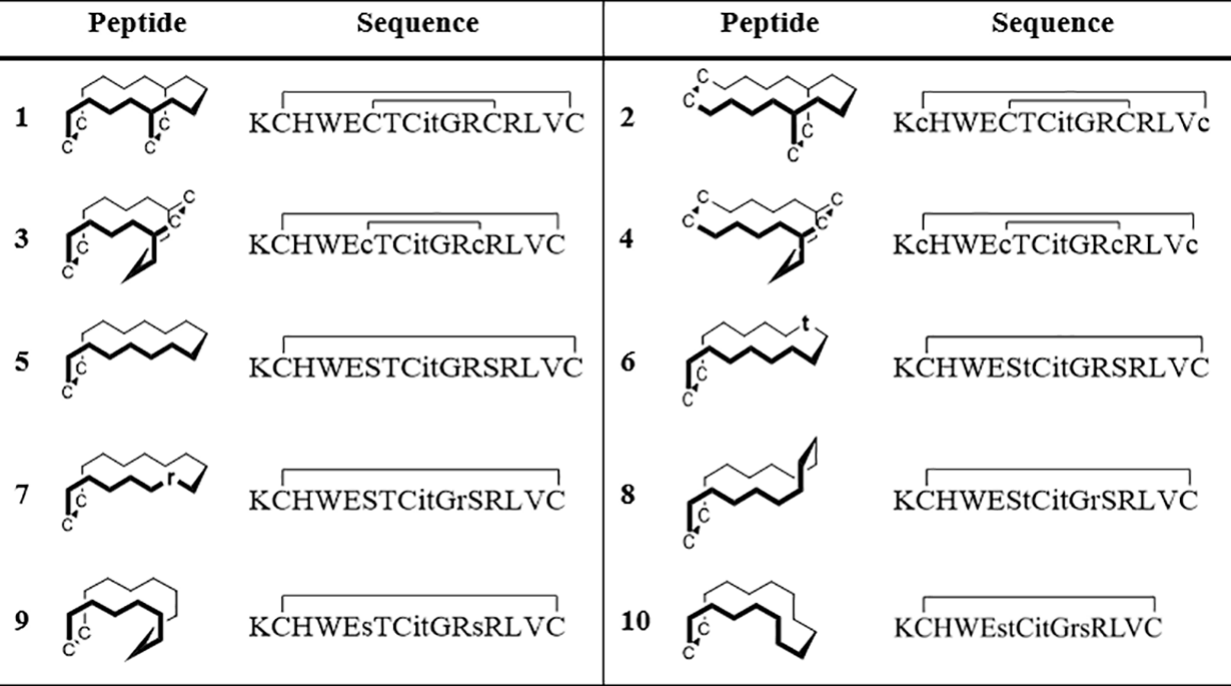
Sequences of the bi-disulfide peptides 1–4 and the mono-disulfide peptides 5–10. They contain one or a pair of d-configurated amino acids and are depicted as simplified zigzag structures in accordance with Fig 1. d-amino acids are denoted by lowercase letters. The directed bending and tethering of peptide backbones results from the combination of the hydrophobic cluster, disulfides, and d-amino acids.

β-hairpin peptides own characteristic conformational properties that render them especially suitable for their use as assembled (also called conformational) epitopes[1]. On one hand, a β-turn as one of the most common, biologically active reverse turn peptide shapes[2–5] can be optimally exposed to the receptor on this framework. On the other hand, the zigzag-like shape of the antiparallel β-sheets, depicted in Figs 1 and 2, creates a 'top' and a 'bottom' face relative to the backbone, each with its unique pattern of amino acid side chains[6]. One side binds to the paratope (antibody) so that the opposite side only plays an indirect role in the recognitions process. The β-hairpin-epitope is therefore literally assembled from non-neighbouring amino acids rather than directly adjacent ones. Consequently, its conformation determines the biological activity, which can be finetuned as well as restrained by chemical methods[7].

Fig 1 shows a group of synthetic hairpins with their respective amino acid sequence, wherein pairs of d-configured amino acids have a characteristic influence on the peptide structure: They create a right-angle kink upward or downward depending on the position of the double-d-amino acid pair and therefore directly affect the orientation of the amino acid functionalities in regard to the hairpin’s top and bottom plane[8]. The high regioselectivity of bi-disulfide formation results from the conformational homogeneity of the peptide secondary structure. What we call shape-persistent is explained by the mutual reinforcing of macrocyclic disulfide ring closure and tight stacking of the amino acid side chains which is then observable by NMR spectroscopy. The stepwise zipper-like oxidative folding of β-hairpins rearranges and complements the established macrocyclic but flexible CCP (cyclic citrullinated peptide) epitope[9]: We set out to bend and constrict it in complementary shapes with the aim to differentiate autoantibody subtypes based on the inscribed conformational (rather than sequential) differences. In this context, rheumatoid arthritis (RA), a common and well-researched inflammatory disease, serves as a role model. It is especially suited, because a multitude of autoantibody subtypes against citrullinated antigens (anti-citrullinated peptide/protein antibodies, ACPA) is generated during the disease course[10]. Their presence and relative titres are indirectly characterised by their antigenic determinants, which accounts for almost all autoantibodies and underlines the importance of peptides in diagnostic applications[11,12]. Synthetic epitopes that mimic the properties of a biologically active protein sequence have the advantage of high reproducibility in regard to quality and quantity because they are not dependent on highly variable biological systems as antigen source. At the same time, their chemical structure can be adapted to the diagnostic or therapeutic needs by posttranslational modifications (e.g. citrullination), the introduction of unnatural building blocks (like d-amino acids), the covalent attachment of desirable moieties (e.g. fatty acids, fluorescence labels, biotin), cyclisation or oligomerisation[11,12].

Citrulline is a non-proteinogenic hydrolysis product of arginine and the hapten of almost all RA epitopes (its molecular structure is depicted in Fig 1)[13]. It constitutes an important posttranslational modification and plays a crucial role in the inflammatory processes, generating a subset of (over-)citrullinated proteins with corresponding antibodies against citrullinated proteins/peptides (ACPAs).[14–16] The so far most potent synthetic peptide antigens are cyclic citrullinated peptides (CCPs), derived from the filament-associated protein filaggrin[9] with a very high serological specificity[14]. The discovery of other antigenic targets like citrullinated fibrinogen[17], alpha-enolase[18], or vimentin[19] added valuable information about the serological status and progression of RA and proved the existence of distinct antibody subtypes (which can readily be explained by the RA disease course[20,21]). Still, the problem solving has mostly been centred on primary structures[22] and their unidimensional variation, in contrast to our modular, pairwise (two-dimensional, compare Figs 1 and 2) modifications, resulting in three-dimensional changes and analyses thereof. Here, we correlate the antibody subtypes with peptide conformations and their unique spatial features, thus defining the conformational epitope landscape[23].

To retain relevant epitopes in the preferred conformation of a β-hairpin, we utilised the Cochran cluster motif[24,25] (module 1, C^1^HWE …RLVC^14^) and added a citrullinated filaggrin sequence[26] (module 2, S^5^TCitGRS^10^) which was subsequently further modified by introducing pairs of directly opposing d-amino acids[8] (module 3) onto this shape-persistent framework. This strategy delivered antibody-selective peptide epitopes (the RA antibody subtypes anti-CCP and anti-Sa could be distinguished, Sa standing for the first probed patient Savoie). The pairwise modification of hairpin amino acids led to the dimerisation of the citrullinated epitopes by deletion of amino acid pairs (module 4)[27], and to the generation of constricted, privileged motifs by introduction of a second disulfide bridge (module 5)[28]. In this work, we show how to combine these synthetic methodologies for creating new β-hairpin conformations. We explain the consecutive selection process for ELISA tests against blood sera and develop approaches for a personalised RA diagnosis with a set of these chosen peptides.

## Materials and methods

### Peptide synthesis and purification

Peptide synthesis was performed on a microwave-assisted peptide synthesizer (*Liberty Blue*, *CEM*). Thereby, a Fmoc-strategy was applied. The experimental details for resin loading, Fmoc-deprotection, amino acid coupling, resin cleavage and disulfide formation can be accessed in the supporting information. Peptides were purified by semi-preparative reversed-phase HPLC on a *Thermo Fisher Ultimate 3000 LC System* [column: ACE5 SuperC18, 150 mm x 10 mm i.d., gradient: 15–30% B in 20 min (A: water + 0.1% TFA, B: acetonitrile + 0.085% TFA), flow rate: 7.00 mL/min].

### Analytical HPLC

Analytical HPLC measurements of all peptides was executed on a *Thermo Fisher Ultimate 3000 LC System* [column: ACE Ultracore 2.5 SuperC18, 150 mm x 2.1 mm i.d., gradient: 10–40% B in 10 min (A: water + 0.1% TFA, B: acetonitrile + 0.085% TFA), flow rate: 0.450 mL/min, see Table A and Figs A to E in S1 File].

### Mass spectrometry

Mass spectra (ESI+) of purified peptides were acquired on a *Thermo Fisher Scientific LTQ-FT Ultra* mass spectrometer). The resolution was set to 100.000. Exact masses of all peptides can be accessed in Table A in S1 File and the high-resolution spectra of bi-disulfides **1**–**4** and the mono-disulfide **2md** in Figs F-J in S1 File.

### NMR measurements and assignment

One- and two-dimensional NMR spectra (1H, TOCSY, NOESY, ^1^H-^13^C-HSQC) were acquired on a Bruker AV600 600 MHz spectrometer at 280 K, 290 K or 300 K in 90% potassium phosphate buffer (50 mM, pH = 3.0) and 10% D_2_O using WATERGATE pulse sequence water suppression. NMR spectra were calibrated to the signal of the internal standard 3-(trimethylsilyl)-2,2,3,3-tetradeuteropropionic acid sodium salt (TSP-d4) that was set to 0.00 ppm. The signal assignment of all peptides was undertaken on the basis of 2D spectra, utilizing TOCSY, NOESY (sequential walk, Fig T in S1 File) and HSQC experiments.

### CD spectroscopy

CD spectroscopy was run on a *Jasco J-810-150S* spectropolarimeter using the following parameters (pH was chosen equal to those of the NMR experiments):

- Peptide concentration: 200 μM in aqueous solution
- Quartz cuvette path length: 0.1 cm
- Wavelengths scanned: 260 nm—190 nm
- Data pitch: 0.5 nm
- Response: 2 s
- Scanning speed: 10 nm/min
- Temperature: 300 K

Data from three consecutive scans were averaged and processed to improve the signal to noise ratio. The mean residue ellipticity (MRE in deg cm^2^ dmol^-1^) was calculated using the equation

$$\theta_{MRE}=\frac{\theta \cdot M_{r}}{c\cdot l\cdot n}$$

where θ is the measured ellipticity, M_r_ the molecular weight of the peptide, c the concentration of the sample, l the pathlength of the cuvette and n the number of peptide bonds.

### Enzyme-linked immunosorbent assay (ELISA)

Peptides were diluted to 10 μg/L in Carbonate/Bicarbonate buffer (50 mm, pH = 9.6). 50 μL of peptide solution (triple determination) were added to each well of a 96 well plate (*DNA-Bind® Surface*, *Corning*). After 2 h, the plate was washed three times with PBS-T (phosphate buffered solution with 0.5% Tween 20) and blocked with 2% BSA in Carbonate/Bicarbonate buffer (100 μL/well) for 16 h at room temperature. After washing three times with PBS-T plates were incubated with either

- a primary antibody (anti-CCP or anti-Sa, stock solution: 200 ru/mL [ru: relative units], diluted 1/20 with 2% BSA in PBS-T, 50 μL/well, polyclonal, human, *Euroimmun* Lübeck) or
- a RA patient’s blood serum, either CCP-positive or CCP-negative (diluted 1/200 with 2% BSA in PBS-T, 50 μL/well)

for 1 h. After washing four times with PBS-T, the plate was incubated with HRP-conjugated anti-human IgG (50 μL/well, 0.3 μg/mL in PBS-T, goat) for 30 min, following washing four times with PBS-T. The binding was visualized by adding 50μL/well 3,3’,5,5’-tetramethylbenzidine (TMB) substrate solution (*Merck*). The blue color reaction was stopped by adding 25μL/well H_2_SO_4_ (5% in water). Optical density (OD) was detected at 450 nm using a *Thermo Scientific Multiskan GO* microplate spectrophotometer. Every ELISA was done three times for each peptide.

The antibody sera mentioned under a) were obtained from *Euroimmun*. The cut-off value was defined as 5 ru/mL by *Euroimmun* via UV/Vis spectroscopy. Measured values for a) are given as relative values normalized to best binding (= highest absorbance) peptide **5** to allow better comparability among all peptides, reproducibility and to account for the inhomogeneity of the different serum batches. Measured absorbances for the utilized sera b) are stated as absolute values. Results of the ELISA experiments are presented as the mean ± SD.

All ELISA experiments utilising blood sera were performed in compliance with the relevant laws and institutional guidelines. Ethical approval was provided by the medical ethics committee of the *Philipps-Universität Marburg*. Collection of human blood sera from both CCP-positive rheumatoid arthritis patients and from the healthy control group has thus been approved by this committee and was executed by medical staff of the *Rheuma-Zentrum Mittelhessen* (Bad Endbach, Germany). Thereby, every participant gave informed consent.

## Results and discussion

Starting from the all-l peptide sequence K^0^C^1^HWEC^5^TCitGRC^10^RLVC^14^ **1** (K^0^ was added for reproducible plate binding in ELISA tests), this study investigates the combination of synthetic modules, encompassing d-configured pairs of amino acids (module 3) with two disulfide bridges (module 5)[28]. This compilation was synthetically challenging as single or pairwise adjacent d-amino acids are known to disrupt secondary structures[29–31] and more than one disulfide bridge often leads to unselective thiol pairing, resulting in oligomers or hard to purify mixtures, especially when no additional orthogonal protecting groups are used[32]. Still, the hydrophobic cluster (module 1) stabilises the hairpin framework, so that we could synthesise all three variants **2** (c1-c14, C5-C10; lowercase letters denote d-amino acids), **3** (C1-C14, c5-c10) and **4** (c1-c14, c5-c10) without the necessity for a complex Cys protecting group strategy. This technical approach of combining folding modules with epitopes yields bi-disulfide peptides which show excellent oxidative folding properties. To the best of our knowledge, the directed bending and tethering of peptide backbones by d-disulfides (Figs 1 and 2) in the presence of other l/d-cysteines has not yet been described.

**Fig 3:**
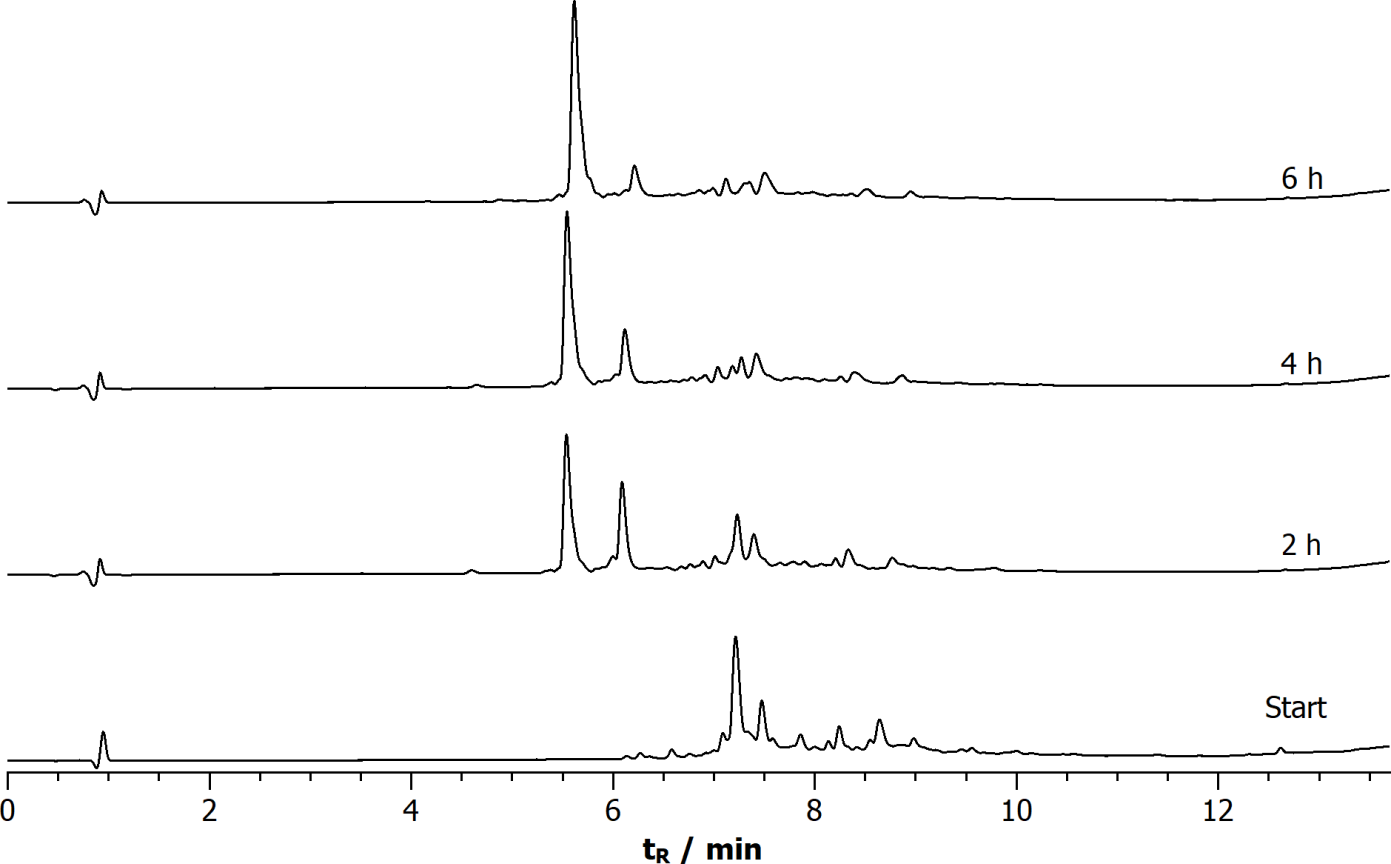
HPLC chromatograms of the oxidative folding process of tetra-d bi-disulfide 4 (c1-c14, c5-c10; c_peptide_ = 1 mg L^-1^). The time periods after which the oxidation process was stopped by addition of TFA is denoted at the right end of the respective chromatograms.

Fig 3 depicts the time-dependent progress of the air oxidation of peptide **4** in (NH_4_)_2_CO_3_ buffer (pH 8.4, c_peptide_ = 1 mg/mL). It is a selected example for a point-to-point reaction, proving the existence of a preferred conformation (the non-availability of a preferred conformation would otherwise result in a complex mixture). Moreover, the consecutive oxidation steps are clearly observable. The first disulfide forms already after one hour (peak at around 6.0 min) and is transformed into the bi-disulfide over time. The reaction is complete after four to six hours and represents a prime example for the so-called framework model, where disulfide oxidation proceeds through a limited number of native-like intermediates[33]. The chromatograms in Fig 3 are representative of all synthesised bi-disulfide derivatives (Figs K-M in S1 File).

**Fig 4:**
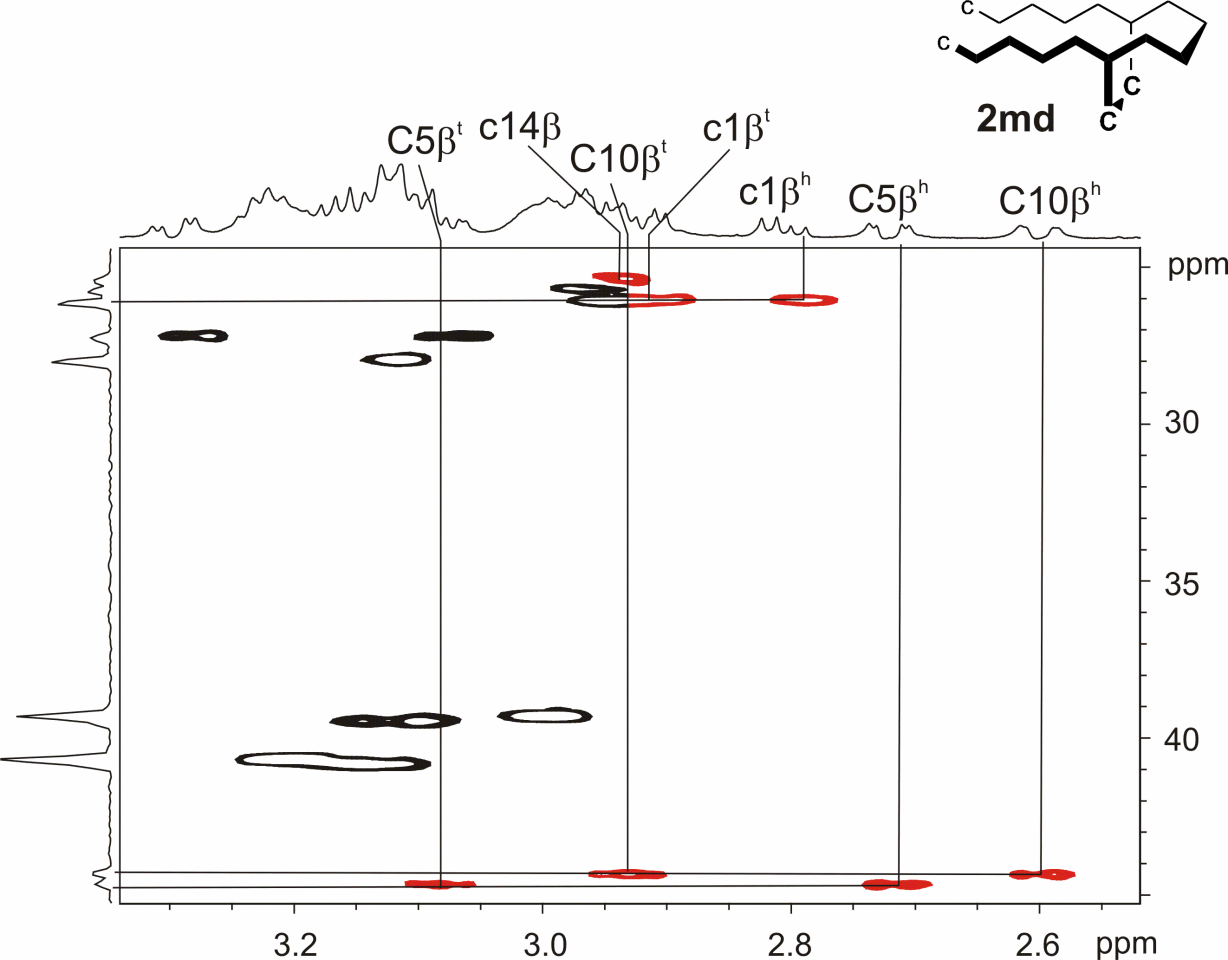
HSQC spectrum excerpt of bi-disulfide peptide 2md (c1/c14,C5-C10). The ^1^*J*_C,H_ correlations between the β-carbons of all cysteines and their corresponding protons are shown. They prove a reduced state for c1 and c14 (~25 ppm, low ^1^H signal dispersion) and a disulfide bridge between C5 and C10 (over 40 ppm, high ^1^H signal dispersion; 600 MHz, 280 K, 50 mm potassium phosphate buffer (pH = 3.0)/D_2_O 9:1).

In the case of peptide **2** (c1-c14,C5-C10), it was possible to isolate the first oxidation product, so that the position of the first formed disulfide could be determined via NMR spectroscopy. After complete assignment of the peptide by means of ^1^H NMR, TOCSY and NOESY spectra, the central cysteine pair of position 5 and 10 emerged as such. The HSQC in Fig 4 provides the necessary experimental data for the corresponding mono-disulfide **2md** (c1/c14, C5-C10): While the β-carbons of c1 and c14 show a chemical shift value of around 25 ppm, the β-carbons of C5 and C10 are shifted downfield to over 40 ppm. The red signals indicate the difference between reduced, low signal dispersion and oxidised, high signal dispersion for the corresponding β-protons. For peptide **4** (c1-c14,c5-c10), we characterised the first closed disulfide bond by quenching the mono-disulfide species with chloroacetamide, subsequent tryptic digestion and LC-MS analysis. As for bi-disulfide **2**, the analytical data indicate that the central c5-c10 disulfide is formed before c1-c14 (Figs A'-D' in S1 File).

The NMR data of all bi-disulfides **1** to **4** substantiate the assumption of their preferred β-hairpin conformation (^1^H-NMR spectra, their comparison and temperature-dependent shift values Δδ/ΔT can be found in Figs N-V in S1 File). They show high signal dispersions in their ^1^H NMR spectra, especially well observable in the amide proton region. This is an unambiguous sign of a highly populated fold, which is tightly tethered by two disulfides even in different stereoconfigurations. The NMR spectra prove that the stereochemistry of the central disulfide bond in position 5 and 10 has more influence on the spatial structure than the terminal one. Chemical shifts among species **3** and **4** with the correlated d-mutation c5-c10 resemble each other far more closely than **2** and **4** with c1-c14. The temperature gradients Δδ/ΔT adopt alternating high and low values, reflecting the alternating H-bonded and non-H-bonded positions within a β-hairpin[34]. Therefore, every species has its own unique β-hairpin shape that addresses a distinct conformational space. Additionally, we measured CD spectra, which can be accessed in the SI (Fig W in S1 File with discussion).

To correlate the peptides’ conformational spaces with biological activities, preliminary ELISA tests of epitopes **1** to **4** against the commercially available autoantibodies anti-CCP and anti-Sa in relation to our best binding mono-disulfide peptide **5** (KC^1^HWES^5^TCitGRS^10^RLVC^14^) gave first valuable insights regarding antibody affinities (a histogram with the results can be found in Fig X in S1 File). They confirm the findings of the conformational analysis, namely that the central double-d mutation exerts more influence than the terminal ones. **3** and **4**, both with c5-c10, show no significant affinity towards either of the RA antibodies. **1** and **2** (C5-C10) indicate absorbance values of around one third compared to best binding epitope **5** (S5,S10). The central l-disulfide restricts the dynamic motions of the turn region that contains citrulline and changes the chemical nature of these positions from two oxygens in the original serines to two sulfurs in cystine. None of the bi-disulfides shows a preference for one or the other autoantibody.

These newly designed folds add to the pool of already synthesised and published peptides[8,26–28]. From this collection, we chose peptides for ELISA tests against sera of RA patients. If only one peptide was applied in an immunoassay, the answer would be a “yes” or “no” for only one type of paratope. If, on the other hand, a set of epitopes is implemented in the test, the data from each one can be compared in relation to each other and a pattern, a kind of bar code (multiple “yes” or “no”), is created for each antibody subtype. By reading out these patterns, paratope profiles can be accessed and new ACPAs identified.

**Fig 5:**
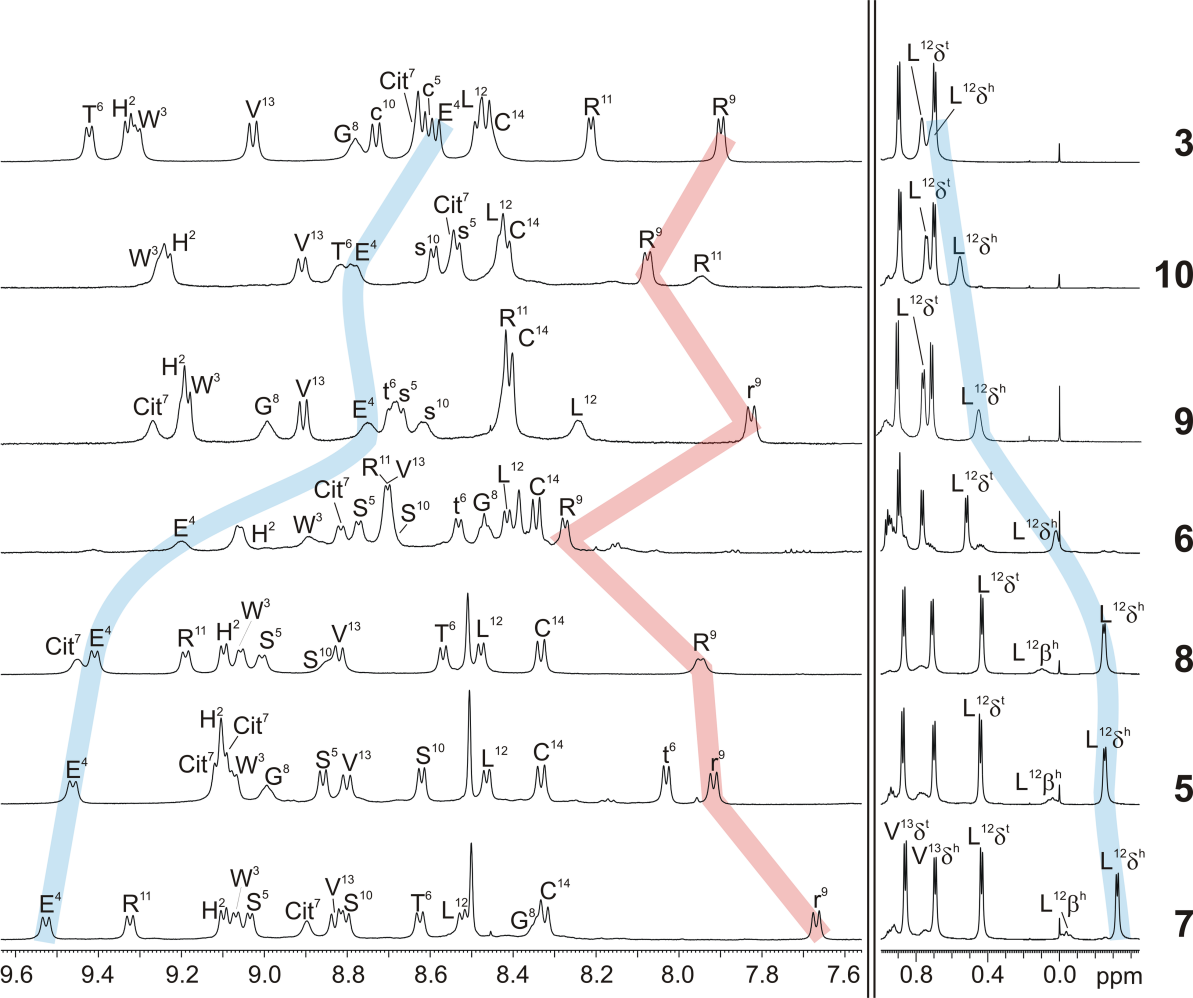
Amide and alkyl regions of the ^1^H NMR spectra of peptides 3 and 5–10. Their different content of pairwise installed d-amino acids corresponds to the respective zigzag shapes depicted in Figs 1 and 2. The peptides are listed according to the characteristic highfield shift of L12-Hδ (right blue ribbon) which identify the completely folded hydrophobic cluster and correlates with the downfield shift of E4-NH (left blue ribbon). R9-NH is in all cases the most shielded amide proton (red ribbon; 600 MHz, 280 K, 50 mm potassium phosphate buffer (pH = 7.0)/D_2_O 9:1).

The selection process was established based on the structural data and the preceding immunoassays against commercially available antibodies. Only peptides with a preferred conformation (indicated by high signal dispersions in NMR) are worth considering, as changes in affinity can be directly connected to structural details. Regarding their biological activity, peptides with no or low affinity against the reference anti-CCP antibodies present promising targets because they complement the good anti-CCP binders. All-l epitope **5** belongs to the latter good binders and was chosen as reference antigen, furthermore the mono-d derivatives **6** (t6) and **7** (r9), the double-d peptides **8** (t6,r9) and **9** (s5,s10), a tetra-d peptide **10** (t6,r9,s5,s10) as well as double-d bi-disulfide **3** (C1-C14, c5-c10; see complete sequences in Fig 2). Relevant sections of the epitopes' ^1^H NMR spectra are depicted in Fig 5. The L12-Hδ and the E4 amide proton are highlighted (blue) as their shifts higher or respectively lower field correlate very well with each other and with the conformational properties of the peptides. They are both determinants for the integrity of the hydrophobic cluster, the main stabilising element of the hairpin. Still, they do not verify a highly populated fold as isolated parameter. Despite differences in these shifts, all epitopes show a high ^1^H NMR signal dispersion in solution, mono-d hairpin **6** (t6) being the exception to the rule with a medium dispersion. The shielding of the R9/r9-NH protons, which only occurs in an intact turn structure, is representative as important indicator for the existence of a global preferred conformation. All in all, these spectra prove the integrity of the β-hairpin secondary structures and demonstrate at the same time the uniqueness of the designed folds.

**Fig 6:**
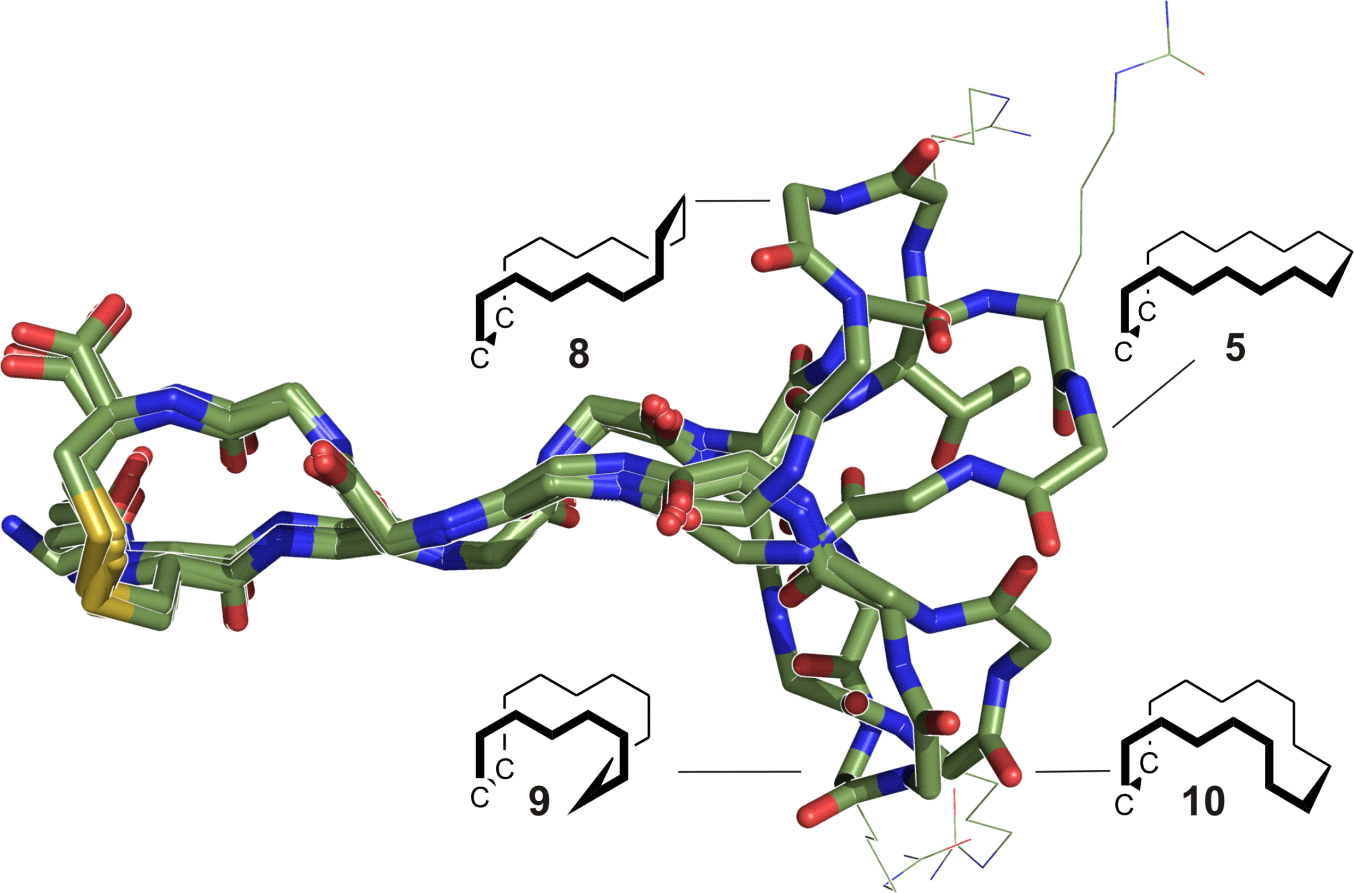
NMR-based computer models of peptides 5 (all-l), 8 (t6,r9), 9 (s5,s10) and 10 (t6,r9,s5,s10) as overlay. The hydrophobic clusters differ only minimally, while the citrulline (shown as thin lines) containing turn exhibits differently kinked geometries, based on the position and amount of d-amino acid pairs. Simplified zigzag structures are depicted at the respective turn.

Together with other spectroscopic details, in particular 2D NMR spectra, we created NMR-based molecular structures. ^3^*J* coupling data and NOEs quantified side chain rotamers about χ_1_ for all amino acids. Four of those structures (corresponding to all-l peptide **5**, double-d peptides **8** and **9** as well as tetra-d peptide **10**) are shown as overlay in Fig 6. They emphasise the right-angle kink and its effect on the conformational properties of the β-hairpins, being representative for mono- and bi-disulfides alike. The terminally positioned hydrophobic cluster is similar to equal among all species, while the turn region, containing citrulline, is bent into different geometries. At the same time, these NMR-based structures point out the right-handed twist of the hairpin backbone.

In ELISA tests, we examined the validity of our test system. The blood sera of a control group of six healthy subjects were screened against the selected antigens **3** and **5** to **10**. They delivered very low absorbance values in ELISA (Fig Y in S1 File) and therefore the important evidence of no false positive results. The otherwise best binders of anti-CCP antibodies, antigen **5** and **8**, showed maximum absorbance values of around 0.40. These data offer a good reference point as they do not differ much among all tested sera.

**Fig 7:**
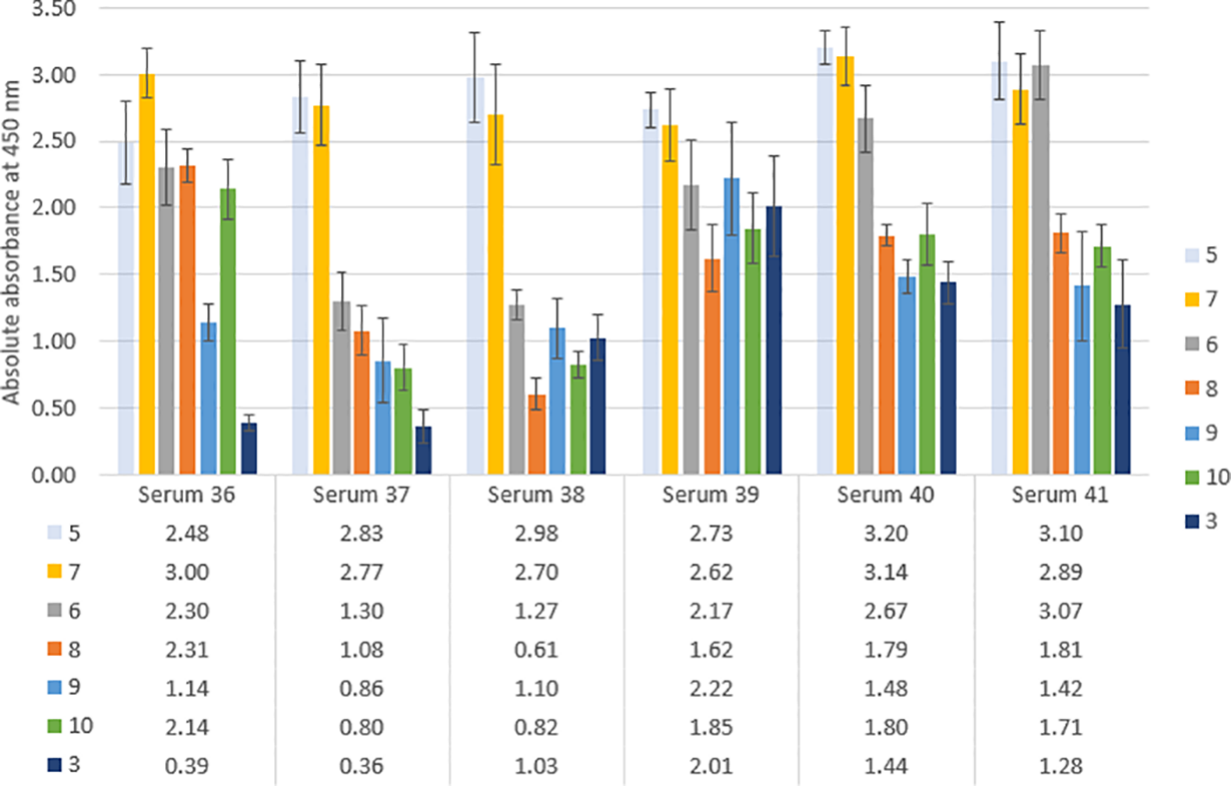
Absolute absorbance values obtained in ELISA for the CCP-positive RA patients. The numbers of the sera have been randomly assigned during their collection. Every patient shows a distinct set of measured absorbances in regard to the utilised epitopes. The order of the peptides is chosen based on the conformational design: first the all-l reference peptide **5**, then the two mono-d peptides **7** (r9) and **6** (t6), afterwards the double-d motifs **8** (t6,r9) and **9** (s5,s10), finally the tetra-d-sequence **10** (t6,r9,s5,s10) as well as the only bi-disulfide **3** (C1-C14,c5-c10).

Subsequently, six sera of CCP-positive patients were tested in ELISA against the described antigens. The results (Fig 7) show diverse patterns for all sera in regard to the different epitopes. On first sight, the well-defined collection of peptide antigens confirms the hypothesis of uncovering differences in antibody profiles, where a single peptide would maximally lead to a statement about the titre of one known paratope. The reference peptide **5** (all-l, transparent blue bar) always showed high absorbance values of 2.50 and higher. The same was true for mono-d peptide **7** (r9, yellow bar). If these two, conformationally very similar peptides adopt high absorbance values, the presence of anti-CCP antibodies can be definitely asserted. Consequently and in compliance with our findings against commercially available sera[8], an early diagnosis could be secured with these positive controls.

Still, this statement could also be made with already available tests and their sequences. That is the reason why the other peptides are of high value, especially (and exclusively) in relation to the affinities of **5** and **7**. Most importantly, the absorbance ratio of the best binders **5**/**7** always added up to 1:1, while all other species didn’t show proportional responses among the sera. E. g. for serum 36, the absorbance ratio of epitope pair **5**/**6** sums up to around 1:1, while in the case of serum 38 it amounted to approximately 1:3, despite **6** exhibiting a similar hairpin conformation as **5** and **7** and generally showing medium to high affinities. On the basis of this finding, it can be assumed that different autoantibody subtypes with different conformational preferences must be present in the tested sera.

Double-d mutant **8** (t6,r9, orange bar) proved to be a weak to medium binder with varying patterns (e.g. serum 38 contains no antibodies that recognise the epitope, but serum 36 a lot of them). Similar results were obtained with double-d hairpin **9** (s5,s10, blue bar), which was less affine for serum 36 in exchange for higher absorbance values for serum 38, and with tetra-d peptide **10** (t6,r9,s5,s10, green bar). A different behaviour was found for bi-disulfide epitope **3** (C1-C14, c5-c10, purple bar: while the sera 36 and 37 did not comprise paratopes, which bind the constricted peptide, medium affinities were observed in the cases of sera 38 to 41.

For completion and comparison of these data, we employed the same set of peptides against CCP-negative patients (meaning those individuals that show pathological signs of RA but do not exhibit a relevant response in the commercial anti-CCP diagnostics). The results of these ELISA tests can be found in Fig Z in S1 File. No false positive results were obtained, and only low absorbance values are generally measured (up to 1.00), though a little bit higher than those of the control group (Fig Y in S1 File). Not a single serum shows an absorbance pattern that would resemble the serological barcodes of the CCP-positive patients. In addition, these patterns are not even completely equal among the CCP-negative individuals. One serum (48) stands out with high absorbance values only for reference peptide **5** (all-l) and mono-d epitope **6** (t6). This exclusive combination, found in a CCP-negative patient serum, is a first hint that the serological barcode approach with a chosen set of peptides could improve the distinguishability from CCP-positive individuals. Still, far more blood sera need to be tested in this regard. The most convincing results in the context of autoantibody recognition patterns have been obtained with the CCP-positive sera (which is to be expected based on the disease course, resulting in a multitude of citrullinated proteins in the synovia [14]).

## Conclusion

Our hairpin restriction technology employed the novel combination of a hydrophobic cluster with a citrullinated epitope, d-amino acid pairs and one or two (differently configured) disulfide bridges. Thus, highly shape-persistent peptide epitopes with unusual conformational features could be generated upon regioselective oxidative folding and analysed in detail. The described modules are readily available tools that are transferable to other epitope designs in autoimmune diagnostics. Our systematic combination of folding does not necessitate the screening of large peptide libraries and it exploits the regioselective oxidative folding of oligo-disulfide epitopes without a time-tested evolutionary refinement. It is therefore complementary to other optimisation approaches of peptide sequences.

The profiles of the blood sera identified by our set of assembled citrullinated epitopes suggest autoantibody subtypes with different conformational preferences. Otherwise, the absorbance ratios between all employed epitope conformations and especially in relation to the best anti-CCP binders **5** and **7** should always be equal. In this context, it is not possible to exactly distinguish between the actual existence of autoantibody subtypes and polyclonality. Still, considering the RA disease course[20,21] and different ACPA studies[14–19] so far, it seems very likely that patients with anti-CCP antibodies in their blood stream also develop a multitude of other autoantibodies against citrullinated proteins in the synovia (which is the definition of a subtype). We could clearly show that our systematic conformational variations of the epitope shape yield immediately visible evidence for these antibody variations (one could coherently call them conformational subtypes), a fine-tuning of paratope variances and distribution. To support that aim, CCP-negative RA patients should be screened against our defined set of conformational epitopes in forthcoming immunoassays to identify unusual patterns and profiles.

## Supporting information

S1 File Additional details of synthetic procedures and detailed analytical data: Results of mass spectrometry and HPLC retention times/chromatograms of synthesised peptides (Table A and Figs A-E in S1 File), high resolution mass spectra (Figs F-J in S1 File), HPLC chromatograms of oxidative folding processes (Figs K-M in S1 File), NMR signal assignment (Tables B-F in S1 File) and ^1^H NMR spectra (Figs N-R in S1 File), comparison of chosen ^1^H NMR spectra (Fig S in S1 File), example for a sequential walk (Fig T in S1 File), determination of ^1^H NMR temperature gradients (Figs U-V in S1 File), CD spectra (Fig W in S1 File), complementary ELISA results (Figs X-Z in S1 File), detailed methodology for thiol alkylation experiments with subsequent trypsin digestion (Figs A'-D' in S1 File) and information about molecular dynamics simulation.
(PDF)
