# Supplementary material for: A set of conformationally well-defined L/D-peptide epitopes provides a serological bar code for autoantibody subtypes
Source: PLoS One. 2018 Aug 3;13(8):e0201735. doi: 10.1371/journal.pone.0201735 (PMC6075753; doi:10.1371/journal.pone.0201735)
Supplement: S1 File — Additional details of synthetic procedures and detailed analytical data: Results of mass spectrometry and HPLC retention times/chromatograms of synthesised peptides (Table A and Figs A-E in S1 File), high resolution mass spectra (Figs F-J in S1 File), HPLC chromatograms of oxidative folding processes (Figs K-M in S1 File), NMR signal assignment (Tables B-F in S1 File) and 1H NMR spectra (Figs N-R in S1 File), comparison of chosen 1H NMR spectra (Fig S in S1 File), example for a sequential walk (Fig T in S1 File), determination of 1H NMR temperature gradients (Figs U-V in S1 File), CD spectra (Fig W in S1 File), complementary ELISA results (Figs X-Z in S1 File), detailed methodology for thiol alkylation experiments with subsequent trypsin digestion (Figs A'-D' in S1 File) and information about molecular dynamics simulation. (PDF) [file pone.0201735.s001.pdf]

Supporting Information

A set of conformationally well-defined L/D-peptide epitopes provides a serological bar code for autoantibody subtypes

Andreas Schrimpf[1], Dörte Brödje[2], Petra Pfefferle[3], Armin Geyer[1]*

1 Department of Chemistry, Philipps-Universität Marburg, Marburg, Germany

2 Department of Medicine, Philipps-Universität Marburg, Marburg, Germany

3 Comprehensive Biomaterial Bank Marburg (CBBMR), Marburg, Germany

* geyer@staff.uni-marburg.de

Peptide Synthesis

Resin Loading

2-Chlorotritylchloride resin (1.60 mmol/g) was loaded with Fmoc-Cys(Trt)-OH by adding the protected amino acid (1.50 eq) and DIPEA (6.00 eq) in DMF (10 mL/g resin) and stirring for 5 h. After washing the resin with DMF, methanol and DCM several times, the resin was treated with a mixture of DCM/methanol/DIPEA (80:15:5) two times for 30 min and washed several times with DMF, methanol and DCM before it was dried under vacuum. The loading of the used resins was estimated to be between 0.50 mmol/g and 0.60 mmol/g by UV-Vis spectroscopy at 289 nm and 300 nm after cleaving the Fmoc protecting group with 20% piperidine in DMF for 20 min.

Solid Phase Peptide Synthesis

Peptides were synthesized on a microwave assisted peptide synthesizer (*Liberty Blue*, *CEM*). Fmoc-strategy was applied. The 2CTC-resins loaded with Fmoc-Cys(Trt)-OH (scheduled quantity: 0.1 mmol, 1.00 eq) ran through the following cycles of Fmoc-deprotection and amino acid coupling:

- **Fmoc-deprotection**: T = 50 °C, $P_{microwave}$ = 30 W, t = 210 s with piperidine (20 w% in DMF, 3.00 mL/deprotection)

- **Amino acid coupling**:
  - for amino acids except Fmoc-Arg(Pbf)-OH:
    T = 50 °C, $P_{microwave}$ = 30 W, t = 600 s with Fmoc-protected amino acid (0.2 M in DMF, 5.00 eq, 2.5 mL/coupling), DIC (0.5 M in DMF, 5.00 eq, 1 mL/coupling) and Oxyma (1 M in DMF, 5.00 eq, 0.5 mL)
  - for Fmoc-Arg(Pbf)-OH:
    1. T = 25 °C, $P_{microwave}$ = 0 W, t = 1500 s
    2. T = 50 °C, $P_{microwave}$ = 35 W, t = 660 s

    with Fmoc-protected amino acid (0.2 M in DMF, 5.00 eq, 2.5 mL/coupling), DIC (0.5 M in DMF, 5.00 eq, 1 mL/coupling) and Oxyma (1 M in DMF, 5.00 eq, 0.5 mL)

Resin Cleavage

Resin cleavage was performed with a mixture of TFA/H_2O/phenol/TIPS (88:4:4:4) for 5 h. Peptides were precipitated from cold diethyl ether (40 mL), washed two to three times with diethyl ether and lyophilized from water.

Disulfide Formation

Crude lyophilized peptides were dissolved in water and the resulting solution was brought to pH = 8.4 with $(NH_4)_2CO_3$ ($c_{Peptide} \approx 1$ mg/mL). The progress of the oxidation was monitored via analytical HPLC. After completion, the solution was lyophilized.

Table A. Peptide sequences, HPLC retention times (chromatograms in Figs A-E) and ESI-MS (exact masses, Figs F-J) results. Lowercase one letter codes represent D-configured amino acid (Cit: L-Citrulline). The experimental details of peptides **5** to **10** have already been described in a recently published work[1].

**Peptide**	**Sequence**	**t_r / min**	**m/z (calculated)**	**m/z (ESI-MS)**
**1**	KCHWECTCitGRCRLVC	6.44	923.9077 $[M+2H]^{2+}$	923.9073 $[M+2H]^{2+}$
**2**	KcHWECTCitGRCRLVc	5.77	923.9077 $[M+2H]^{2+}$	923.9083 $[M+2H]^{2+}$
**2md**	KcHWECTCitGRCRLVc	6.72	924.9155 $[M+2H]^{2+}$	924.9177 $[M+2H]^{2+}$
**3**	KCHWEcTCitGRcRLVC	5.88	923.9077 $[M+2H]^{2+}$	923.9082 $[M+2H]^{2+}$
**4**	KcHWEcTCitGRcRLVc	5.35	923.9077 $[M+2H]^{2+}$	923.9079 $[M+2H]^{2+}$
**5**	KCHWESTCitGRSRLVC	5.49	908.9383 $[M+2H]^{2+}$	908.9379 $[M+2H]^{2+}$
**6**	KCHWEStCitGRSRLVC	5.56[1]	908.9383 $[M+2H]^{2+}$	908.9407 $[M+2H]^{2+}$
**7**	KCHWESTCitGrSRLVC	5.65[1]	908.9383 $[M+2H]^{2+}$	908.9387 $[M+2H]^{2+}$
**8**	KCHWEStCitGrSRLVC	5.66[1]	908.9383 $[M+2H]^{2+}$	908.9384 $[M+2H]^{2+}$
**9**	KCHWEsTCitGRsRLVC	5.47[1]	908.9383 $[M+2H]^{2+}$	908.9416 $[M+2H]^{2+}$
**10**	KCHWEstCitGtsRLVC	5.70[1]	908.9383 $[M+2H]^{2+}$	908.9377 $[M+2H]^{2+}$

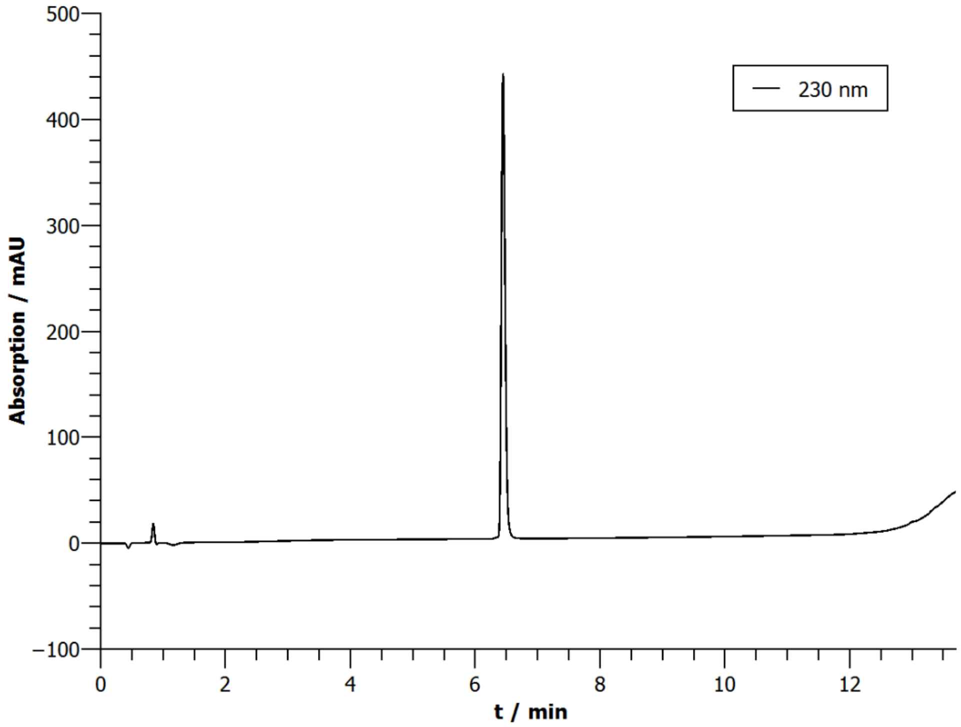

Fig A. Chromatogram of bi-disulfide peptide **1** (all-L, C1-C14, C5-C10).

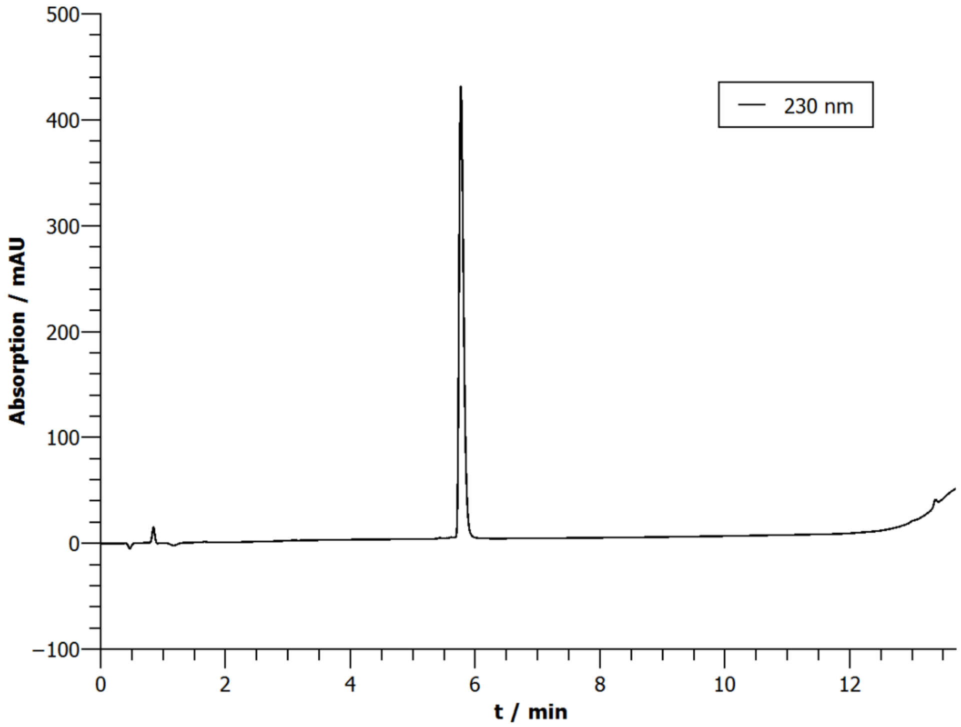

Fig B. Chromatogram of bi-disulfide peptide **2** (c1-c14, C5-C10).

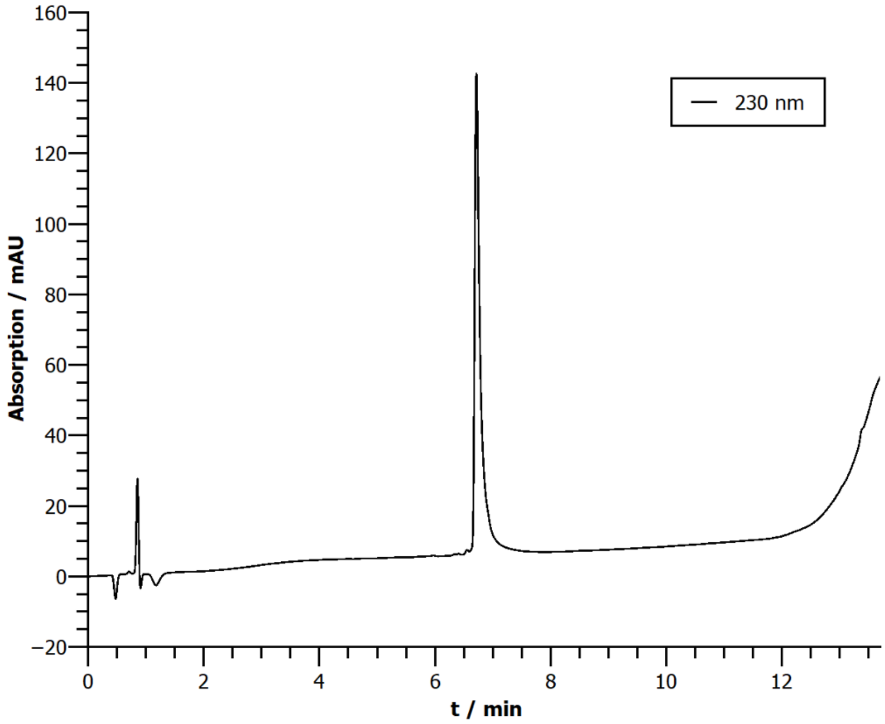

Fig C. Chromatogram of mono-disulfide peptide **2md** (c1/c14, C5-C10).

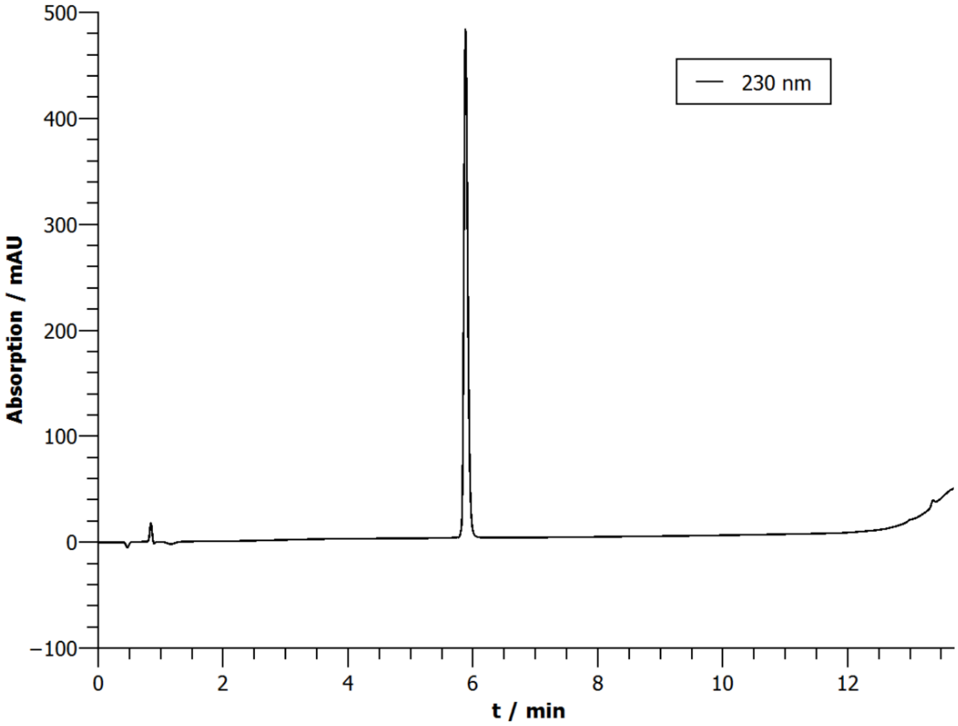

Fig D. Chromatogram of bi-disulfide peptide **3** (C1-C14, c5-c10).

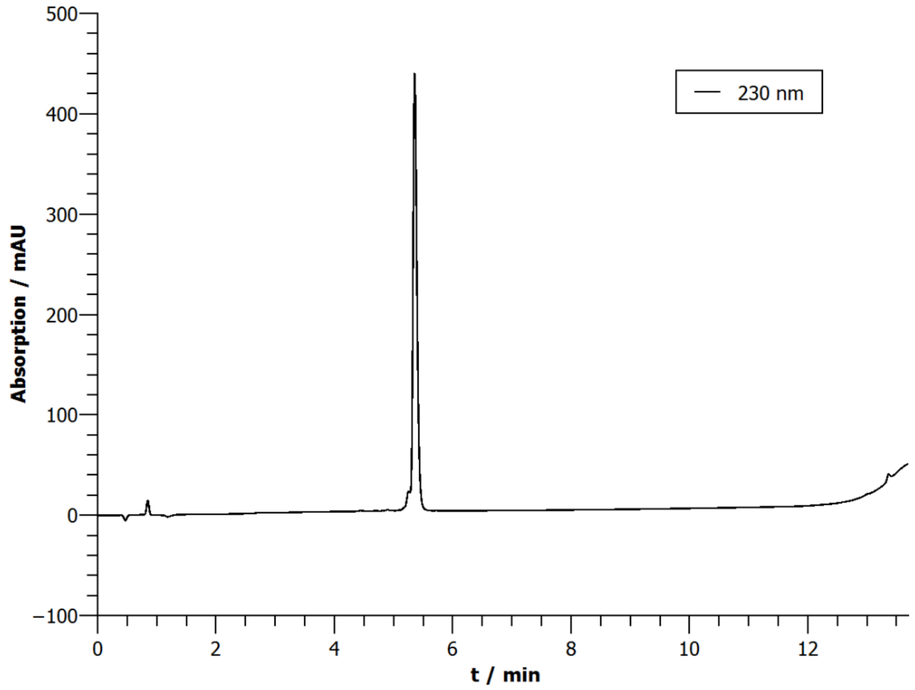

Fig E. Chromatogram of bi-disulfide peptide **4** (c1-c14, c5-c10).

High resolution mass spectra of the bi-disulfides 1 to 4 and mono-disulfide 2md

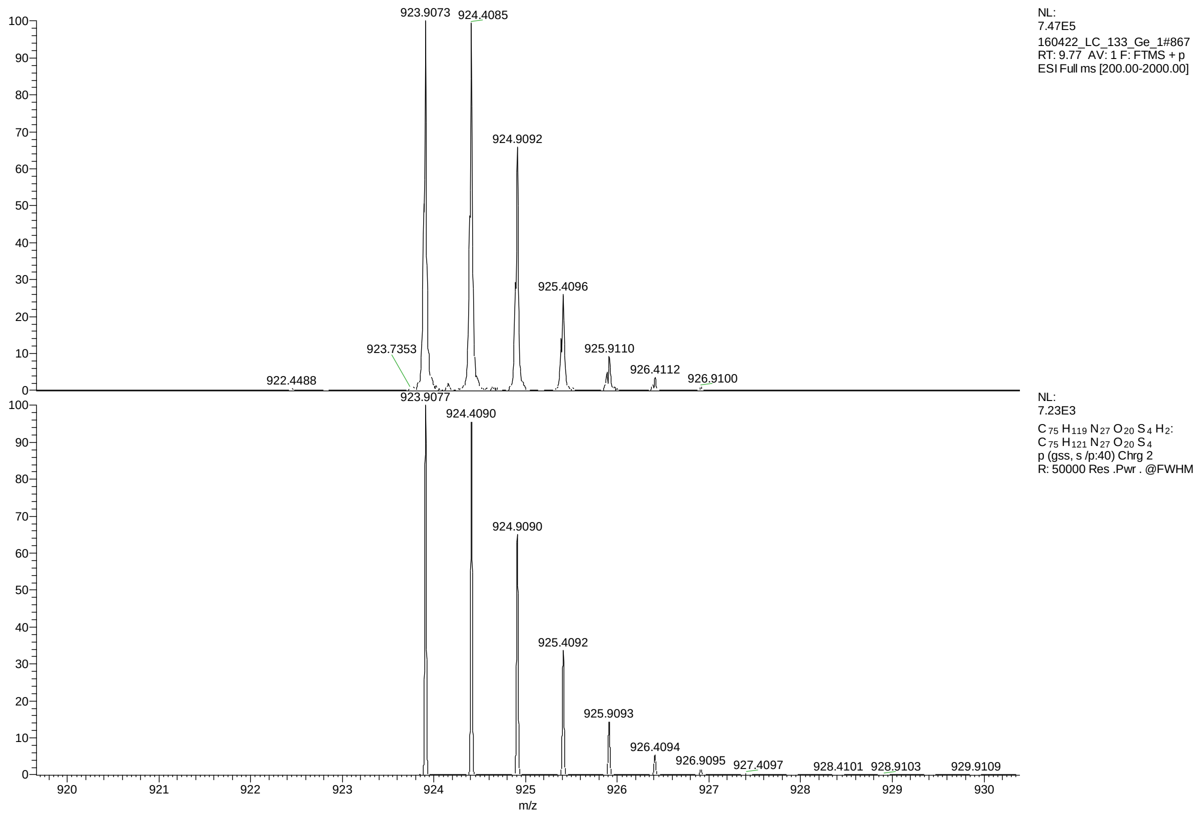

Fig F. HR mass spectrum of bi-disulfide **1** (C1-C14, C5-C10).

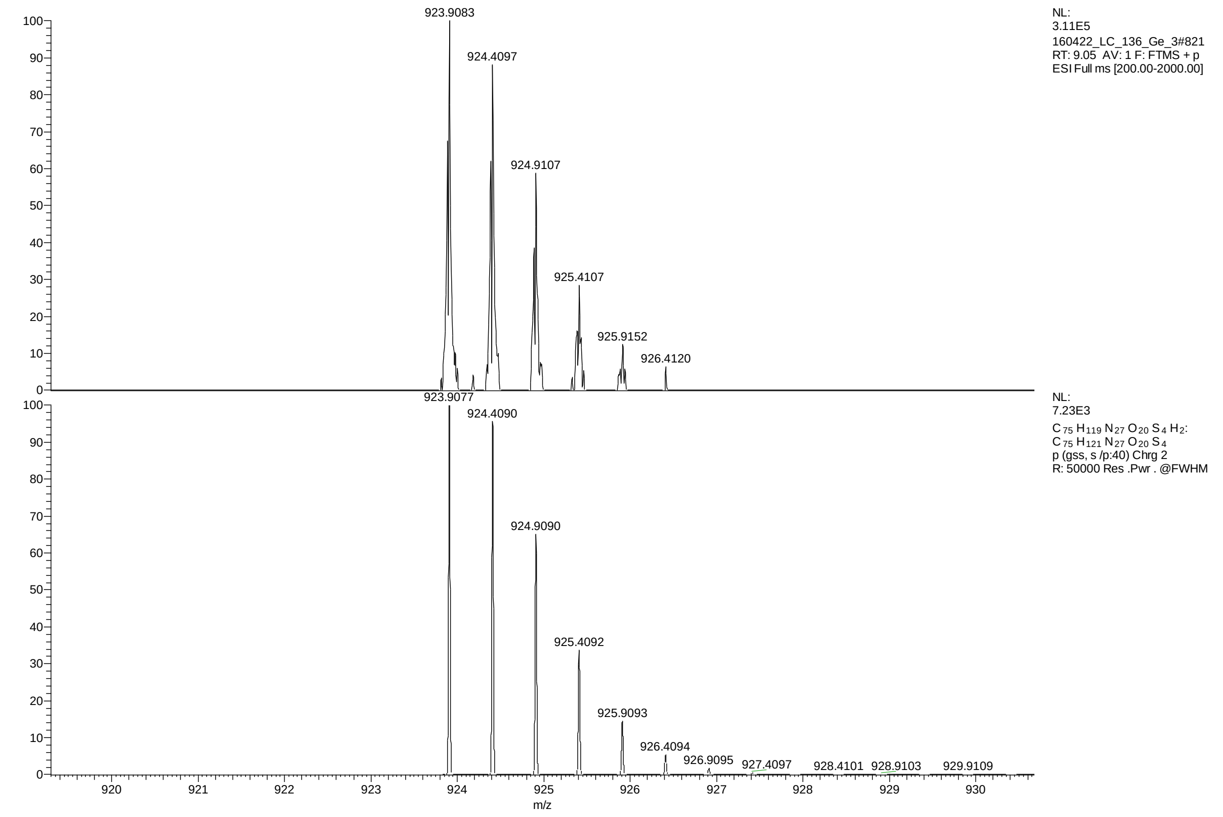

Fig G. HR mass spectrum of bi-disulfide **2** (c1-c14, C5-C10).

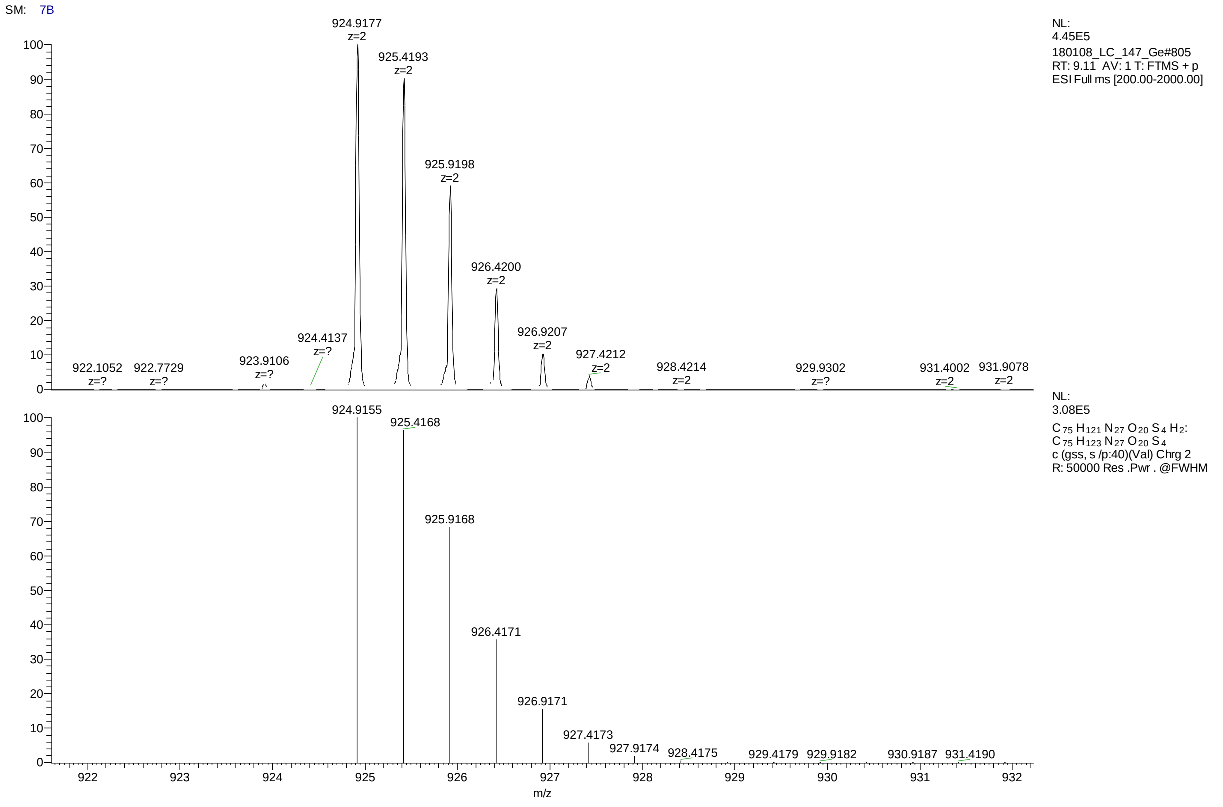

Fig H. HR mass spectrum of bi-disulfide **2md** (c1/c14, C5-C10).

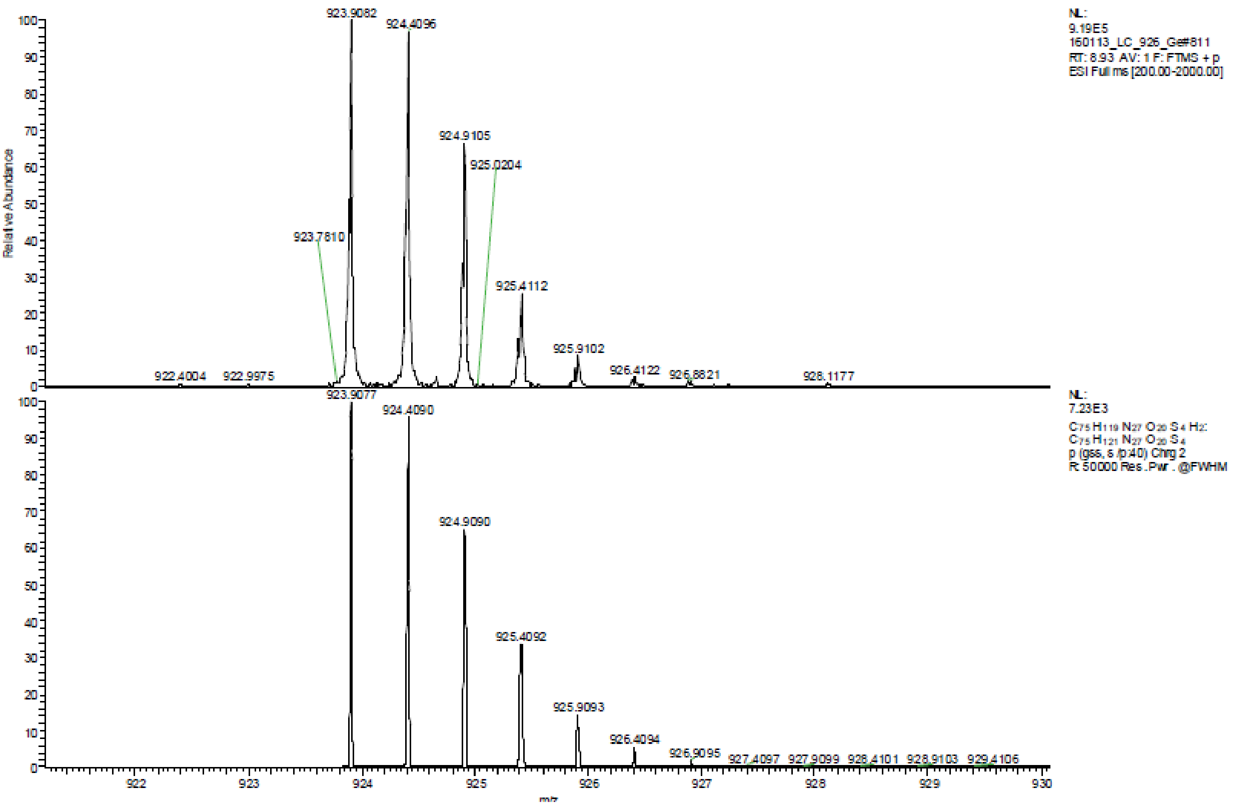

Fig I. HR mass spectrum of bi-disulfide **2** (C1-C14, c5-c10).

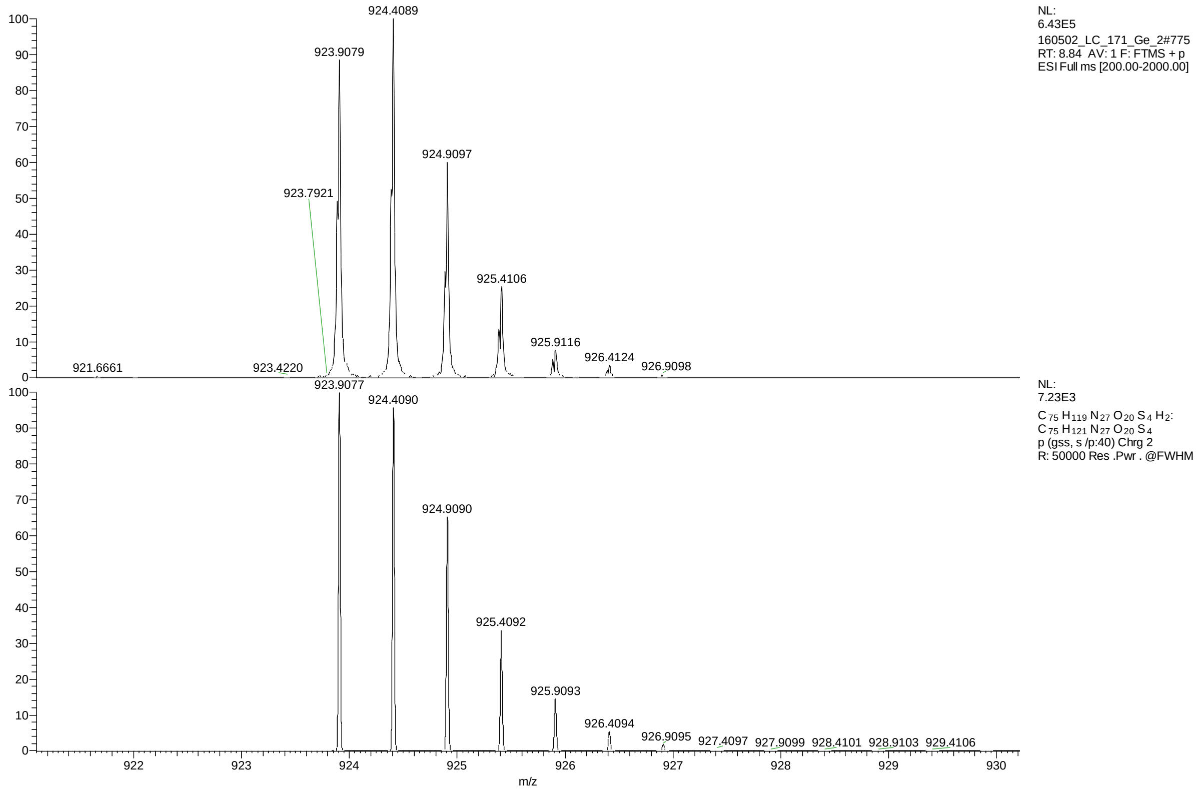

Fig J. HR mass spectrum of bi-disulfide **2** (c1-c14, c5-c10).

HPLC chromatograms for the oxidative folding process of the bi-disulfides

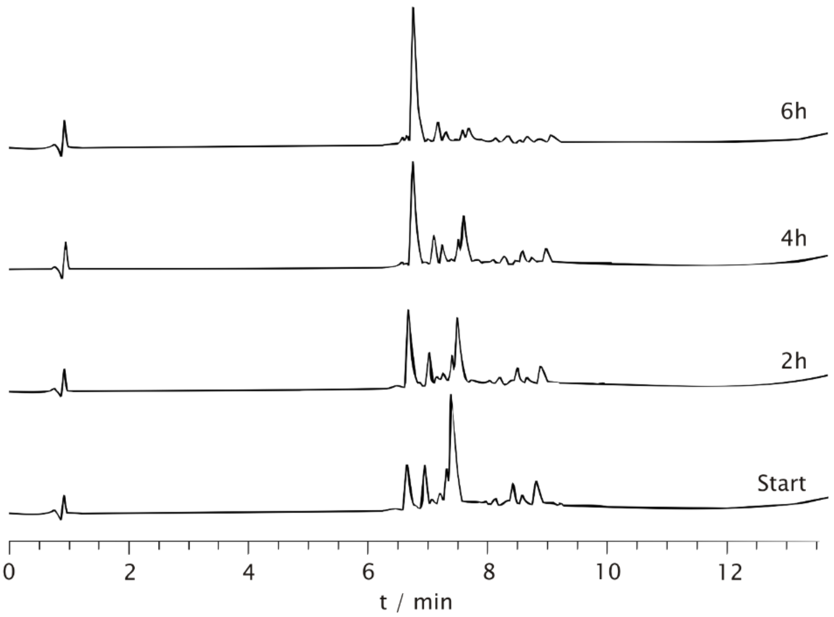

Fig K. HPLC chromatograms of the oxidative folding process of all-L bi-disulfide **1** (c1-c14, C5-C10). The time periods after which the oxidation process was stopped by addition of TFA is denoted at the right end of the respective chromatograms.

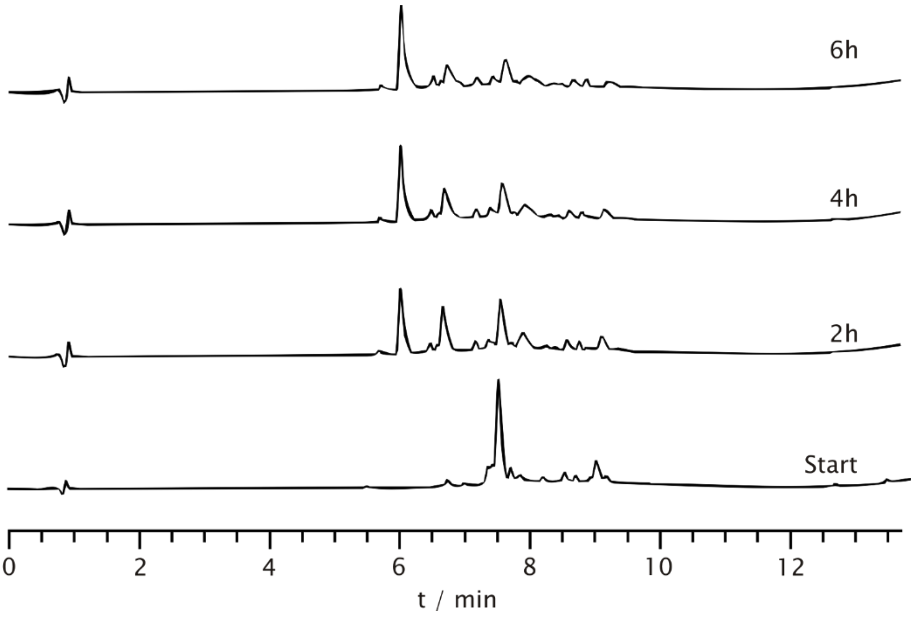

Fig L. HPLC chromatograms of the oxidative folding process of double-D bi-disulfide **2** (c1-c14, C5-C10). The time periods after which the oxidation process was stopped by addition of TFA is denoted at the right end of the respective chromatograms.

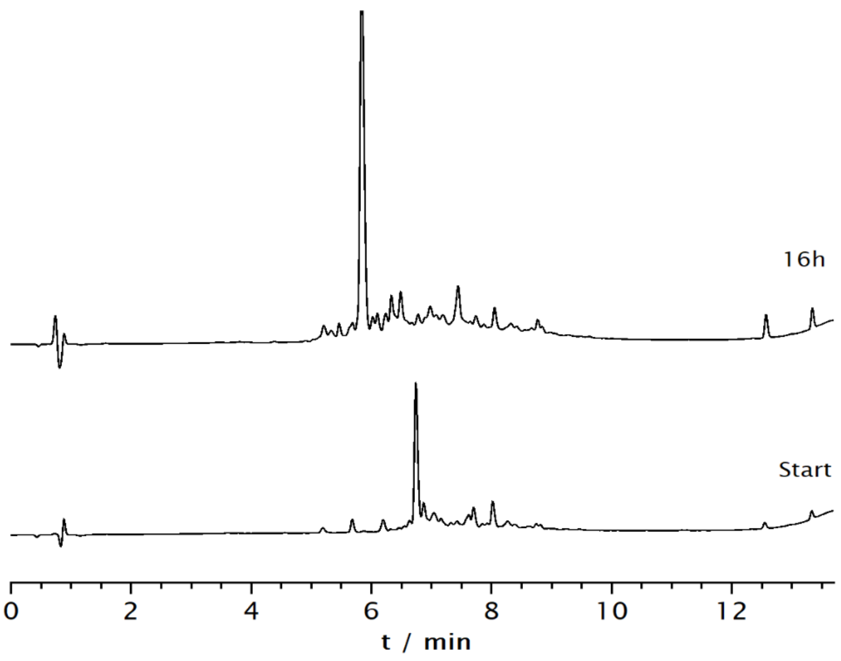

Fig M. HPLC chromatograms for double-D bi-disulfide **3** (c1-c14, C5-C10). The time periods after which the oxidation process was stopped by addition of TFA is denoted at the right end of the respective chromatograms.

Peptide 1

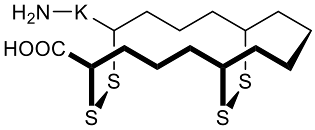

H-**K^{0}-C^{1}-H^{2}-W^{3}-E^{4}-C^{5}-T^{6}-Cit^{7}-G^{8}-R^{9}-C^{10}-R^{11}-L^{12}-V^{13}-C^{14}**-OH

Table B. ^{1}H NMR signal assignment for bi-disulfide peptide **1** (600 MHz, 280 K, 50 mM potassium phosphate buffer (pH = 7.0)/D_2O 9:1).

Amino acid	δ(α) / ppm	δ(β) / ppm	δ(γ) / ppm	δ(other) / ppm	δ(NH) / ppm
**Lys^{0}**	4.09	1.95	1.50	δ: 1.75, ε: 3.03	-
**Cys^{1}**	5.46	3.16, 2.62	-	-	n/a
**His^{2}**	4.89	3.31, 3.20	-	aromatic: δ^{2}: 7.14, ε^{1}: 8.49	9.10
**Trp^{3}**	4.94	3.10	-	aromatic: 2: 7.27, 1(NH): 10.25, 4: 7.12, 7: 7.45, 5: 6.93, 6: 7.18	9.13
**Glu^{4}**	4.85	2.13, 1.88	2.32	-	9.38
**Cys^{5}**	5.61	3.09, 2.68	-	-	9.28
**Thr^{6}**	4.45	4.09	1.24	-	8.76
**Cit^{7}**	3.87	2.00, 1.81	1.59, 1.54	δ: 3.19, 3.13 ε(NH): 6.55	9.65
**Gly^{8}**	3.93, 3.46	-	-	-	8.65
**Arg^{9}**	4.68	1.88	1.65, 1.54	δ: 3.22 ε(NH): 7.33	7.91
**Cys^{10}**	5.70	2.90, 2.73	-	-	9.01
**Arg^{11}**	4.78	1.79, 1,67	1.46	δ: 3.06, 2.89, ε(NH): 7.25	9.15
**Leu^{12}**	4.51	1.31, 0.30	0.78	δ: 0.43, -0.19	8.63
**Val^{13}**	4.00	1.35	0.89, 0.73	-	9.03
**Cys^{14}**	4.70	3.07, 2.86	-	-	8.37

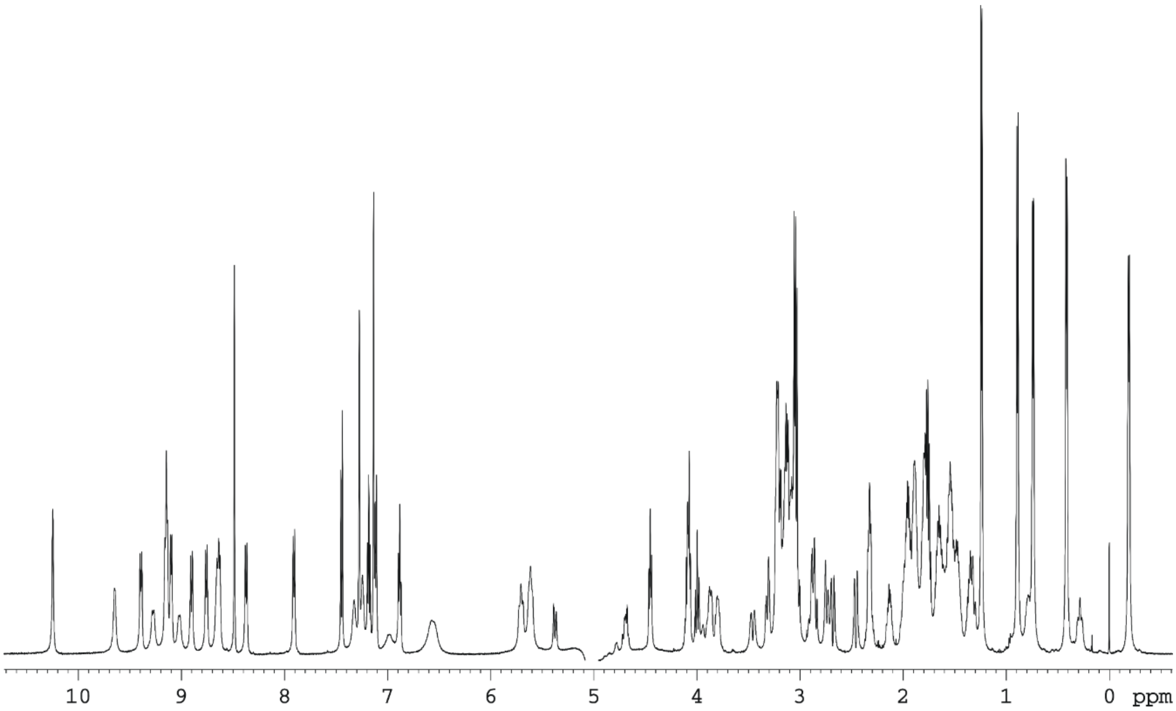

Fig N. ^{1}H NMR spectrum of bi-disulfide peptide **1** (600 MHz, 280 K, 50 mM potassium phosphate buffer (pH = 7.0)/D_2O 9:1).

Peptide 2 (bi-disulfide)

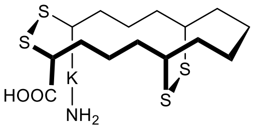

H-**K^{0}-c^{1}-H^{2}-W^{3}-E^{4}-C^{5}-T^{6}-Cit^{7}-G^{8}-R^{9}-C^{10}-R^{11}-L^{12}-V^{13}-c^{14}**-OH

Table C. ^{1}H NMR signal assignment for bi-disulfide peptide **2** (600 MHz, 280 K, 50 mM potassium phosphate buffer (pH = 7.0)/D_2O 9:1).

**Amino acid**	**δ(α) / ppm**	**δ(β) / ppm**	**δ(γ) / ppm**	**δ(other) / ppm**	**δ(NH) / ppm**
**Lys^{0}**	4.03	1.96	1.49	δ: 1.74, ε: 3.01	-
**cys^{1}**	5.36	3.21, 3.00	-	-	n/a
**His^{2}**	4.87	3.32, 3.20	-	aromatic: δ^{2}: 6.87, ε^{1}: 7.99	8.71
**Trp^{3}**	5.00	3.08	-	aromatic: 2: 7.25, 1(NH): 10.24, 4: 7.15, 7: 7.45, 5: 6.92, 6: 7.33	9.00
**Glu^{4}**	4.86	2.12, 1.96	2.31, 2.24		9.44
**Cys^{5}**	5.59	3.08, 2.71	-	-	9.24
**Thr^{6}**	4.45	4.08	1.24	-	8.76
**Cit^{7}**	3.89	1.99, 1.81	1.57	δ: 3.16, ε(NH): 6.52	9.64
**Gly^{8}**	4.02, 3.52	-	-	-	8.69
**Arg^{9}**	4.70	1.89	1.65, 1.54	δ: 3.22, ε(NH): 7.32	7.92
**Cys^{10}**	5.75	2.93, 2.72	-	-	9.00
**Arg^{11}**	4.76	1.81	1.68, 1.45	δ: 3.14 ε(NH): 7.25	9.16
**Leu^{12}**	3.86	1.27, 0.31	0.76	δ: 0.42, 0.09	8.48
**Val^{13}**	4.16	1.61	0.87, 0.71	-	8.84
**cys^{14}**	4.61	3.04, 2.84	-	-	8.27

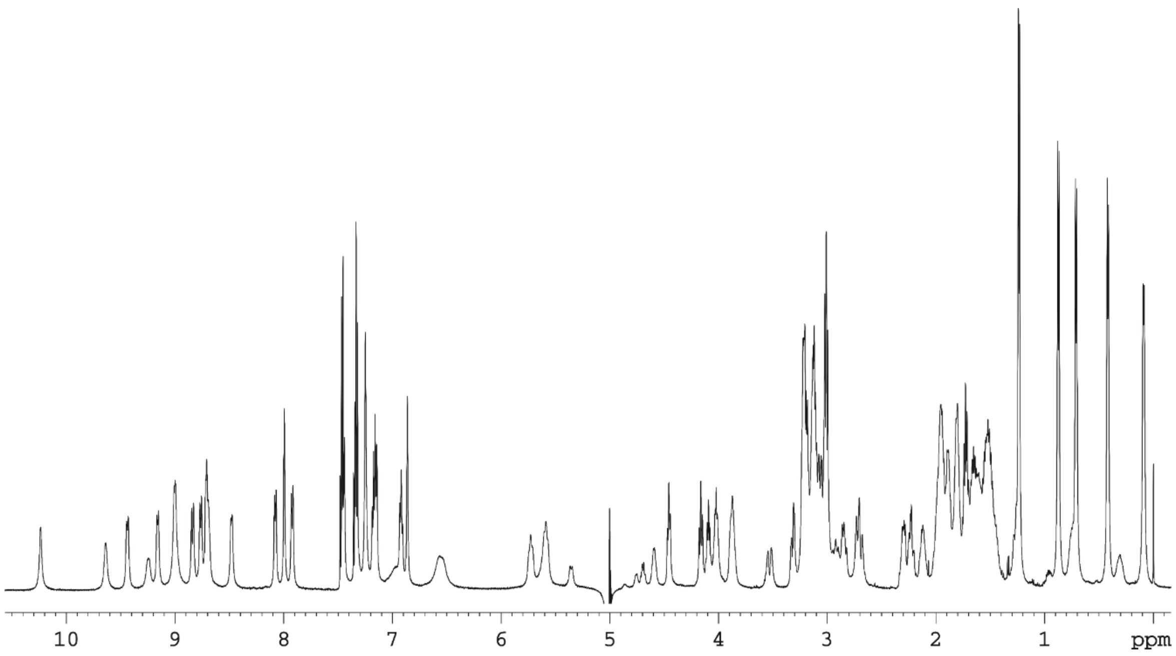

Fig O. ^{1}H NMR spectrum of bi-disulfide peptide **2** (600 MHz, 280 K, 50 mM potassium phosphate buffer (pH = 7.0)/D_2O 9:1).

Peptide 2md (mono-disulfide)

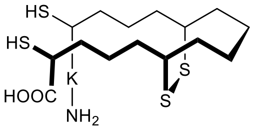

H-**K**^{0}-**c**^{1}-**H**^{2}-**W**^{3}-**E**^{4}-**C**^{5}-**T**^{6}-**Cit**^{7}-**G**^{8}-**R**^{9}-**C**^{10}-**R**^{11}-**L**^{12}-**V**^{13}-**c**^{14}-OH

Table D. ^{1}H NMR signal assignment for bi-disulfide peptide **2** (600 MHz, 300 K, 50 mM potassium phosphate buffer (pH = 3.0)/D_2O 9:1).

Amino acid	δ(α) / ppm	δ(β) / ppm	δ(γ) / ppm	δ(other) / ppm	δ(NH) / ppm
**Lys^{0}**	4.10	1.96	1.47	δ: 1.70, ε: 3.00	-
**cys^{1}**	4.53	2.91, 2.80	-	-	8.75
**His^{2}**	4.92	3.29, 3.07	-	aromatic: δ^{2}: 7.26, ε^{1}: 8.64	8.79
**Trp^{3}**	5.00	3.11	-	aromatic: 2: 7.21, 1(NH): 10.24, 4: 7.22, 7: 7.45, 5: 6.97, 6: 7.18	8.69
**Glu^{4}**	4.72	2.06, 1.89	2.37		9.10
**Cys^{5}**	5.44	3.08, 2.71	-	-	9.01
**Thr^{6}**	4.42	4.08	1.22	-	8.60
**Cit^{7}**	3.93	1.98, 1.80	1.55	δ: 3.12, ε(NH): n/a	9.37
**Gly^{8}**	4.13, 3.62	-	-	-	8.59
**Arg^{9}**	4.64	1.87	1.65, 1.56	δ: 3.22, ε(NH): 7.18	7.90
**Cys^{10}**	5.51	2.92, 2.59	-	-	8.89
**Arg^{11}**	4.65	1.80	1.61, 1.46	δ: 3.13 ε(NH): 7.11	8.84
**Leu^{12}**	4.16	1.36. 0.90	0.99	δ: 0.43, 0.39	8.41
**Val^{13}**	4.16	2.09	0.90, 0.81	-	8.62
**cys^{14}**	4.50	2.94	-	-	8.16

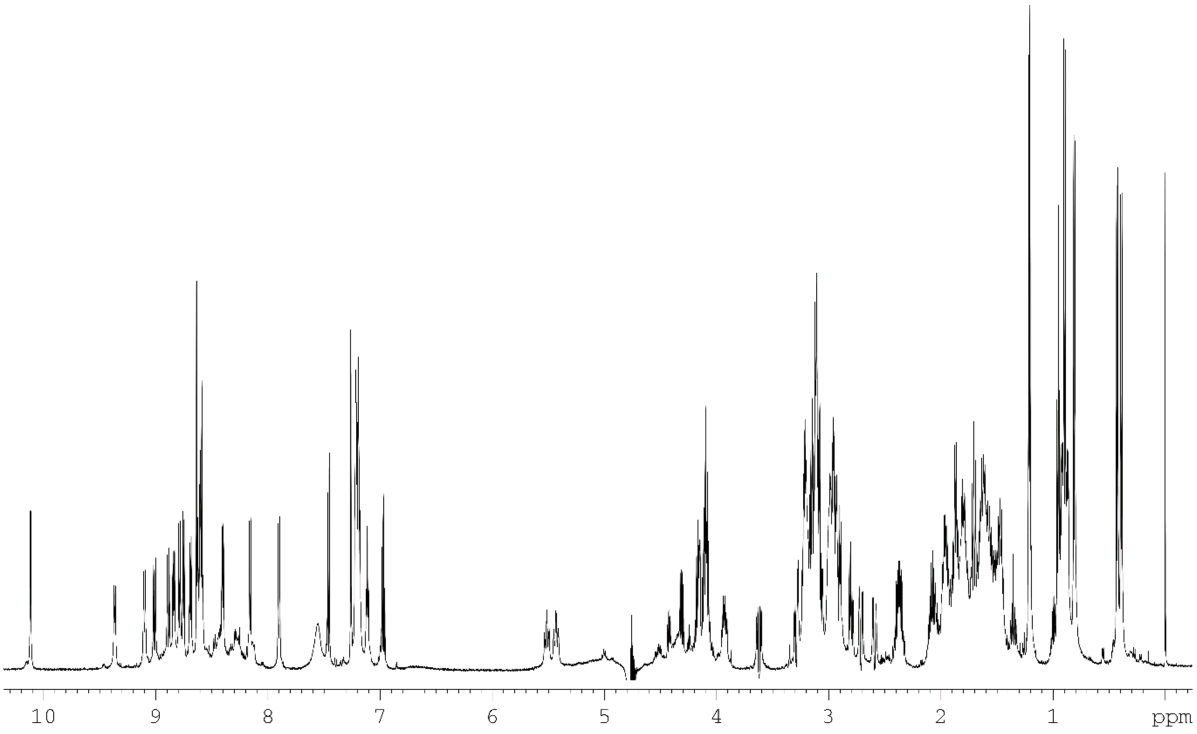

Fig P. ^{1}H NMR spectrum of bi-disulfide peptide **2** (600 MHz, 280 K, 50 mM potassium phosphate buffer (pH = 7.0)/D_2O 9:1).

Peptide 3

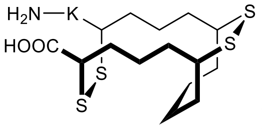

H-**K^{0}-C^{1}-H^{2}-W^{3}-E^{4}-c^{5}-T^{6}-Cit^{7}-G^{8}-R^{9}-c^{10}-R^{11}-L^{12}-V^{13}-C^{14}**-OH

Table E. ^{1}H NMR signal assignment for bi-disulfide peptide **1** (600 MHz, 280 K, 50 mM potassium phosphate buffer (pH = 7.0)/D_2O 9:1).

**Amino acid**	**δ(α) / ppm**	**δ(β) / ppm**	**δ(γ) / ppm**	**δ(other) / ppm**	**δ(NH) / ppm**
**Lys^{0}**	4.09	1.96	1.51	δ: 1.76, ε: 3.05	-
**Cys^{1}**	5.46	3.16, 2.62	-	-	n/a
**His^{2}**	5.03	3.25	-	aromatic: δ^{2}: 7.10, ε^{1}: 8.44	9.33
**Trp^{3}**	4.90	3.08	-	aromatic: 2: 6.71, 1(NH): 9.91, 4: 7.19, 7: 6.91, 5: 6.94, 6: 6.96	9.31
**Glu^{4}**	4.54	1.89, 1.71	2.28, 2.20	-	8.58
**cys^{5}**	5.70	2.96	-	-	8.62
**Thr^{6}**	4.89	4.60	1.22	-	9.42
**Cit^{7}**	4.05	1.79	1.51, 1.46	δ: 3.08 ε(NH): 6.46	8.63
**Gly^{8}**	4.06, 3.98	-	-	-	8.78
**Arg^{9}**	4.87	2.01	1.82, 1.70	δ: 3.22 ε(NH): 7.29	7.89
**cys^{10}**	5.79	2.90, 2.71	-	-	8.73
**Arg^{11}**	3.98	1.60, 1.46	1.20, 1.03	δ: 3.06, 2.89, ε(NH): 7.17	8.21
**Leu^{12}**	4.51	1.76	1.46	δ: 0.77, 0.70	8.49
**Val^{13}**	4.06	1.45	0.90, 0.70	-	9.03
**Cys^{14}**	4.78	3.13, 2.91	-	-	8.46

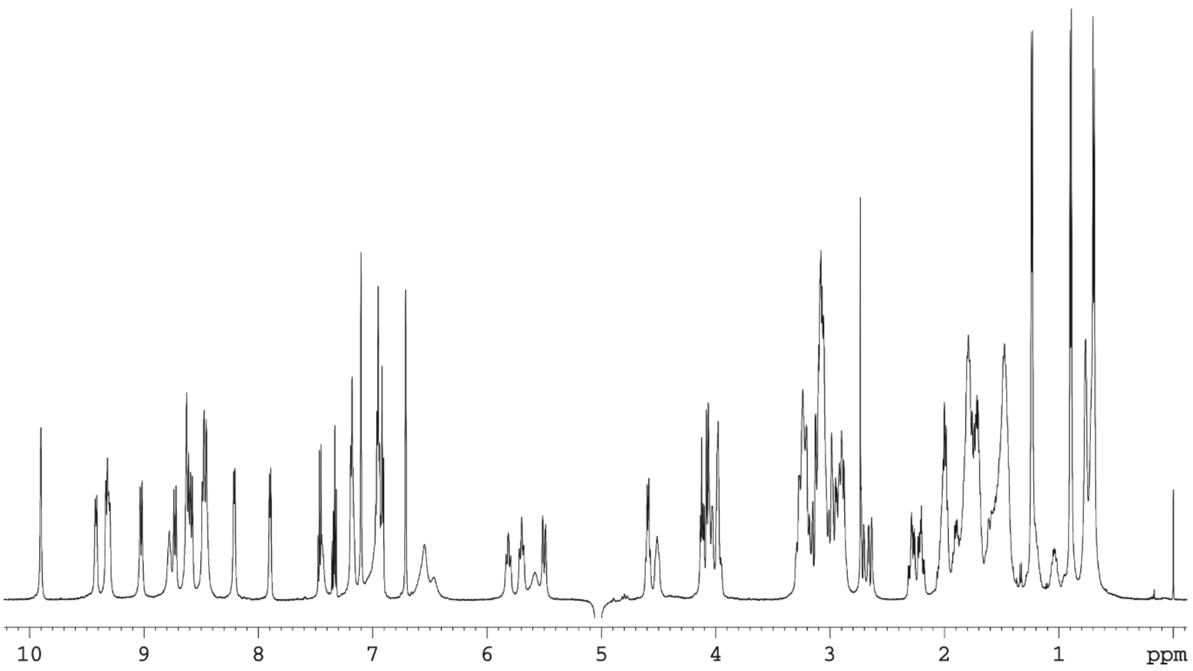

Fig Q. ^{1}H NMR spectrum of bi-disulfide peptide **3** (600 MHz, 280 K, 50 mM potassium phosphate buffer (pH = 7.0)/D_2O 9:1).

Peptide 4

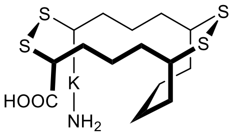

H-**K**^{0}-**c**^{1}-**H**^{2}-**W**^{3}-**E**^{4}-**c**^{5}-**T**^{6}-**Cit**^{7}-**G**^{8}-**R**^{9}-**c**^{10}-**R**^{11}-**L**^{12}-**V**^{13}-**c**^{14}-OH

Table F. ^{1}H NMR signal assignment for bi-disulfide peptide **1** (600 MHz, 280 K, 50 mM potassium phosphate buffer (pH = 7.0)/D_2O 9:1).

Amino acid	δ(α) / ppm	δ(β) / ppm	δ(γ) / ppm	δ(other) / ppm	δ(NH) / ppm
**Lys^{0}**	4.07	2.00	1.53	δ: 1.75,<br>ε: 3.05	-
**cys^{1}**	5.57	3.26, 2.98	-	-	n/a
**His^{2}**	4.90	3.39, 3.20	-	aromatic:<br>δ^{2}: 6.78, ε^{1}: 7.99	8.84
**Trp^{3}**	4.96	3.17		aromatic:<br>2: 6.76,<br>1(NH): 9.91,<br>4: 7.33, 7: 6.93,<br>5: 7.03, 6: 6.99	9.07
**Glu^{4}**	4.64	1.94, 1.81	2.30, 2.21		8.76
**cys^{5}**	5.73	2.97	-	-	8.64
**Thr^{6}**	4.90	4.56	1.22	-	9.37
**Cit^{7}**	4.08	1.80	1.55, 1.46	δ: 3.09,<br>ε(NH): 6.46	8.61
**Gly^{8}**	3.94, 3.06	-	-	-	8.89
**Arg^{9}**	4.90	2.03, 1.85	1.71	δ: 3.22,<br>ε(NH): 7.44	7.92
**cys^{10}**	5.85	2.91, 2.73	-	-	8.75
**Arg^{11}**	4.03	1.67, 1.52	1.23, 1.06	δ: 3.10, 2.89<br>ε(NH): 7.21	8.27
**Leu^{12}**	4.60	1.70	1.70	δ: 1.00	8.49
**Val^{13}**	4.23	1.58	0.85, 0.64	-	8.95
**cys^{14}**	4.69	3.13, 2.82	-	-	8.27

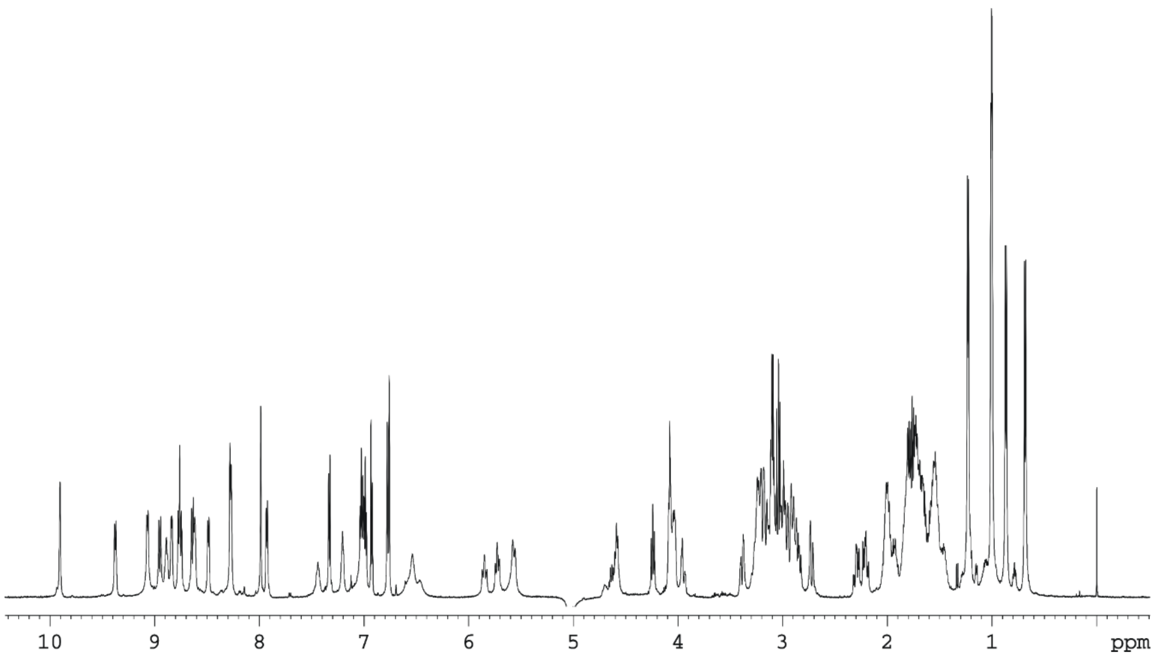

Fig R. ^{1}H NMR spectrum of bi-disulfide peptide **3** (600 MHz, 300 K, 50 mM potassium phosphate buffer (pH = 3.0)/D_2O 9:1).

The ^{1}H NMR spectra of peptides 5 to 10 have already been described in a recently published work[1].

NMR comparison

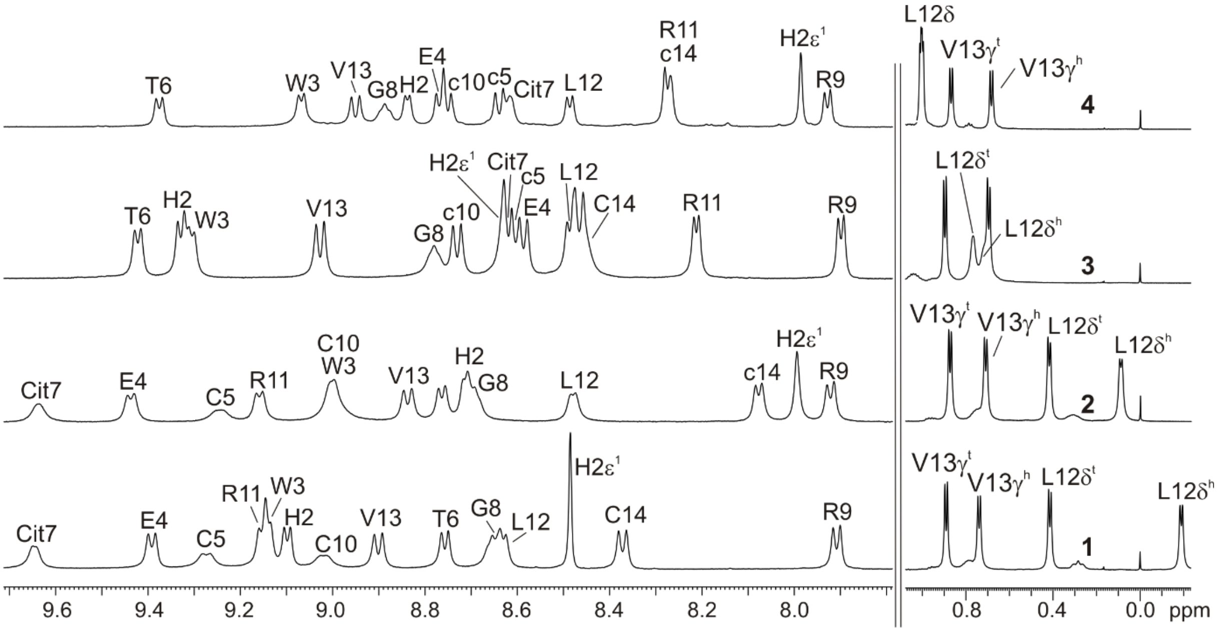

Fig S. Amide regions of the bi-disulfide peptides **1** to **4** with differently configured (L/D) disulfide pairs (600 MHz, 280 K, 50 mM potassium phosphate buffer (pH = 7.0)/D_2O 9:1).

Sequential Walk

The correct sequence was proven by backbone sequential walks. The amide protons in a hairpin peptide show a weak NOE contact to their own Hα, while they show an intense NOE contact to the Hα of the previous amino acid in the sequence. Fig T shows this sequential walk exemplary for bi-disulfide **3** (C1-C14, c5-c10).

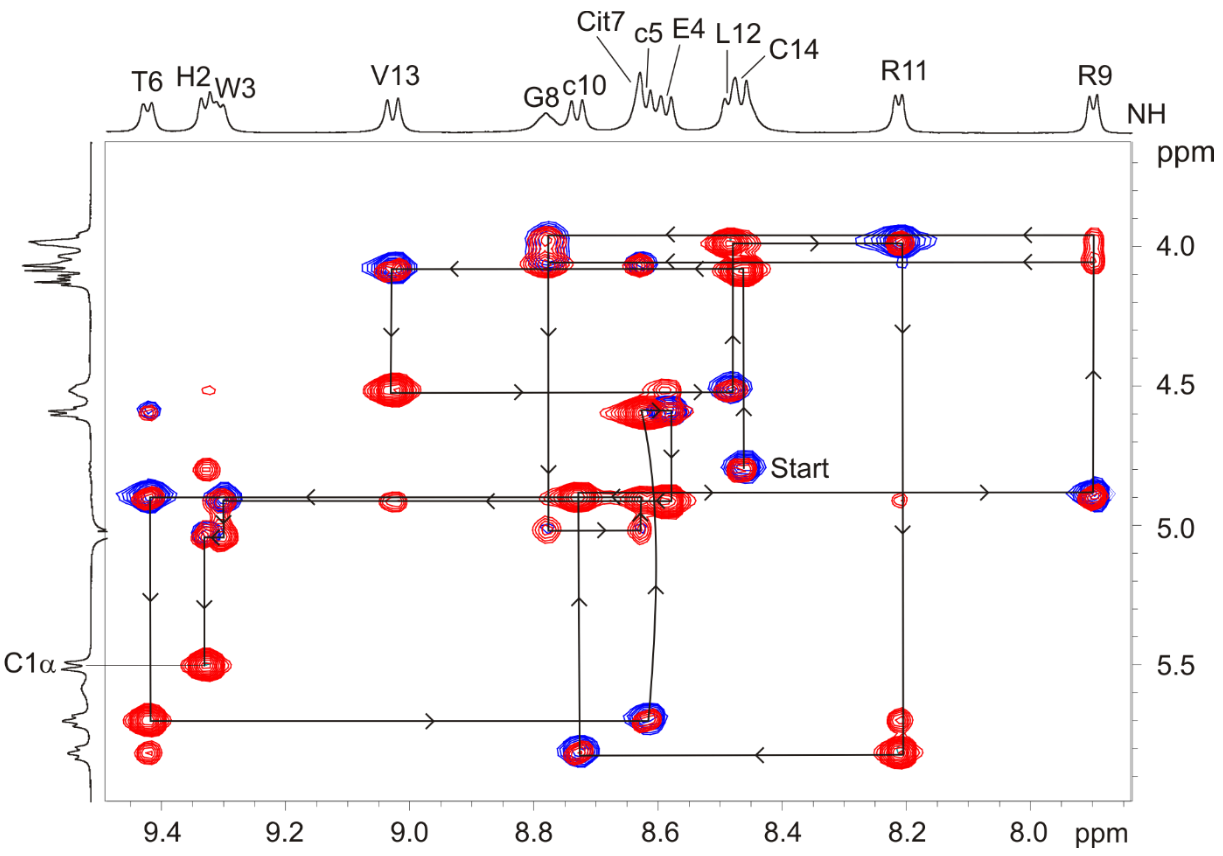

Fig T. Overlay of the NH-Hα region of the NOESY (red) and TOCSY (blue) spectrum of bi-disulfide **3** (C1-C14, c5-c10) (600 MHz, 300 K, 50 mM potassium phosphate buffer (pH = 7.0)/D_2O 9:1). The sequential walk is indicated with arrows, starting from the C-terminal C14-NH.

Temperature Gradients of the Bi-disulfides

^{1}H NMR spectra were measured at 280 K, 290 K and 300 (exemplary depicted for bi-disulfide **3** (C1-C14, c5-c10) in Fig U). The change of shift for every NH signal was determined as ppb/K. Amide protons in hydrogen bonds are less dependent on temperature than those exposed to solvent. A large dispersion of those values and the typical alternating higher and lower

dependencies for a rigid hairpin motif prove the integrity of the structure. Fig V shows the peptides' LEWIS structures with the temperature gradient values in ppb/K.

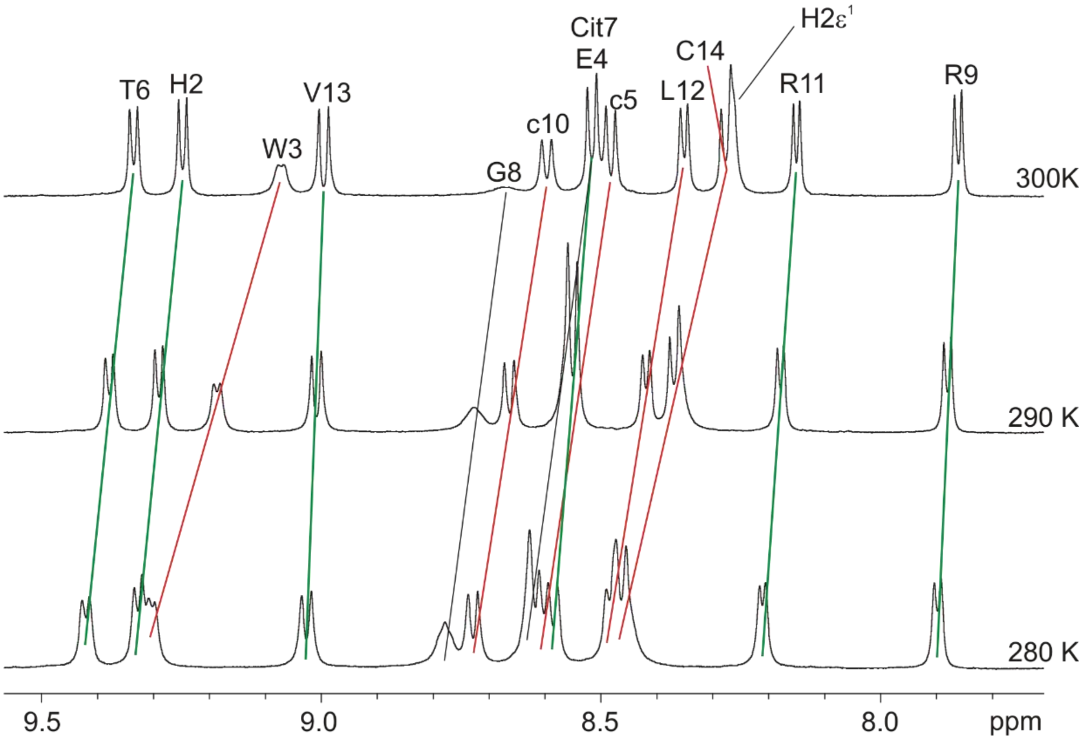

Fig U. Depiction of the amide region of the ^{1}H NMR spectra for bi-disulfide **3** (C1-C14, c5-c10) measured at 280, 290 and 300 K (600 MHz, 50 mM potassium phosphate buffer (pH = 7.0)/D_2O 9:1). The lines indicate the temperature dependent shift of the amide signals.

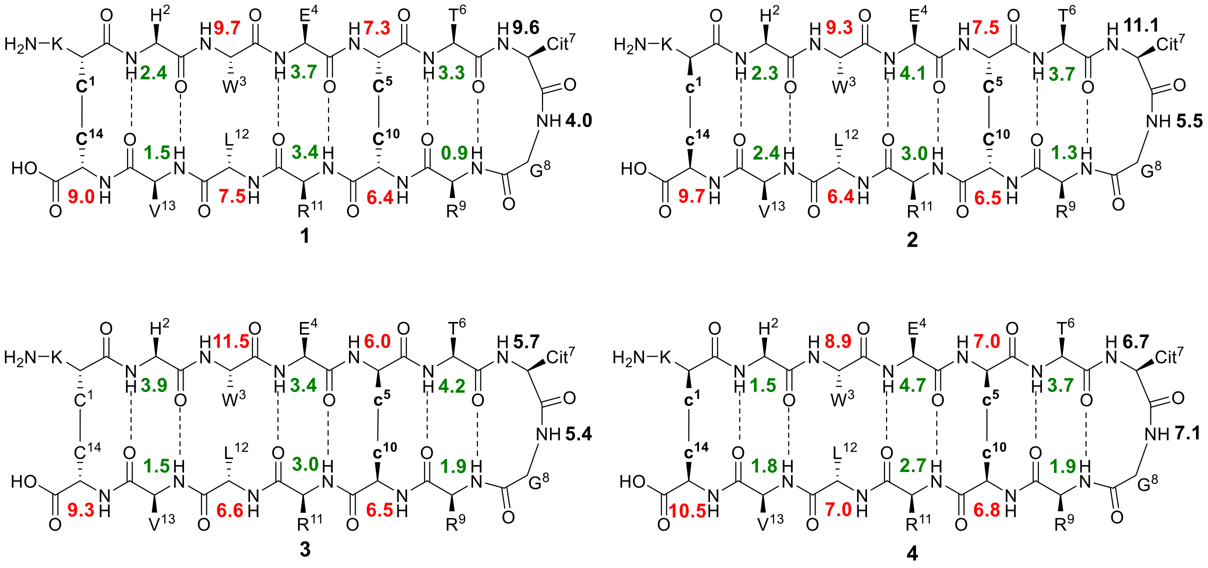

Fig V. Temperature gradients in ppb/K for bi-disulfide peptides **1** to **4**.

CD spectroscopy

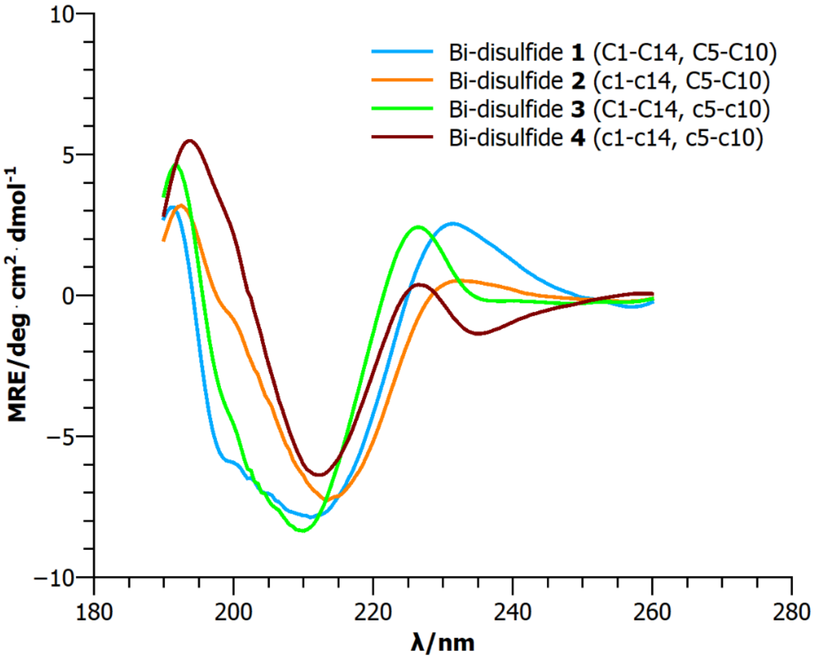

Fig W. CD spectra of the bi-disulfide peptides **1-4**.

The all-L bi-disulfide **1** (C1-C14,C5-C10, light blue) shows a CD spectrum with a maximum at 231 nm and an unusually broad minimum at around 211 nm. The spectrum of bi-disulfide **2** (c1-c14,C5-C10, orange) provides a typical spectrum expected for an antiparallel β-sheet peptide with a slight maximum at 233 nm and a defined but less intense minimum at 213 nm. Double-D bi-disulfide **3** (C1-C14, c5-c10, green) exhibits an overall similar spectrum as **1** with a hypsochromic shift of the maximum to 226 nm. The data of tetra-D peptides **4** (c1-c14, c5-c10, dark red) yield a so far not observed minimum at 235 nm that merges into a maximum at 226 nm. The second minimum at 212 nm turns out less intensive than for the other bi-disulfides. All in all, the measured spectra complement the NMR data that are discussed in detail in the manuscript. They indicate unusual β-hairpin conformations influenced by the different combinations of D/L-configured disulfide pairs.

Enzyme-linked immunosorbent assay (ELISA)

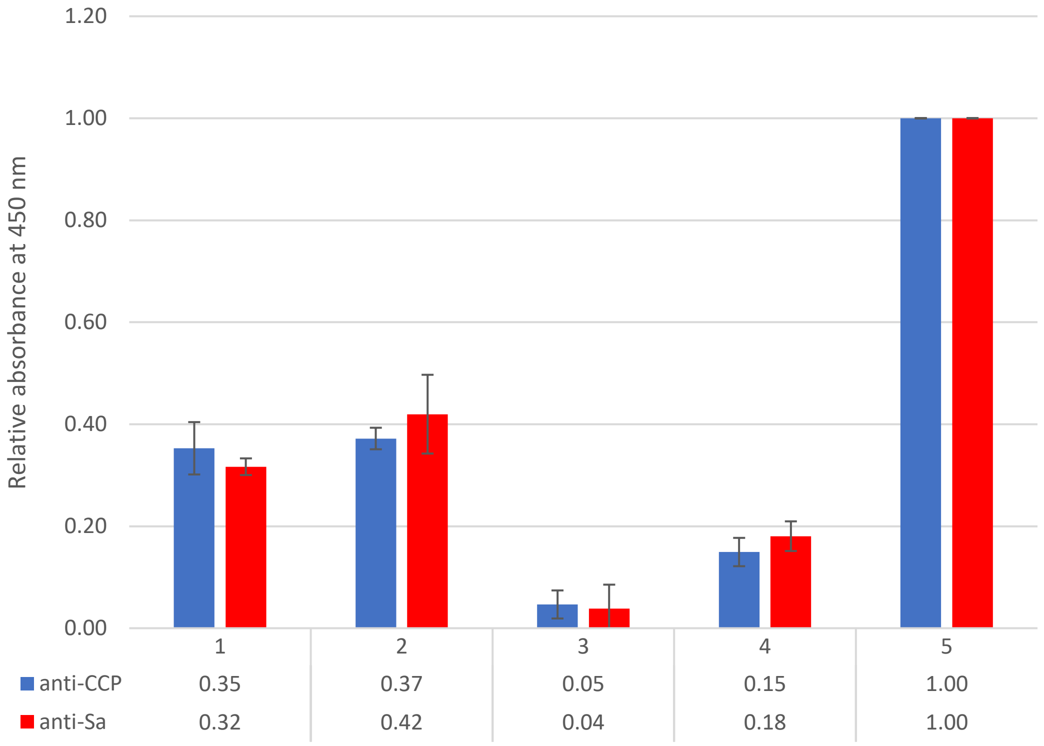

Fig X. ELISA results of the differently configured bi-disulfides **1** to **4** relative to the best binding peptide **5** when utilising anti-CCP or anti-Sa antibodies, respectively.

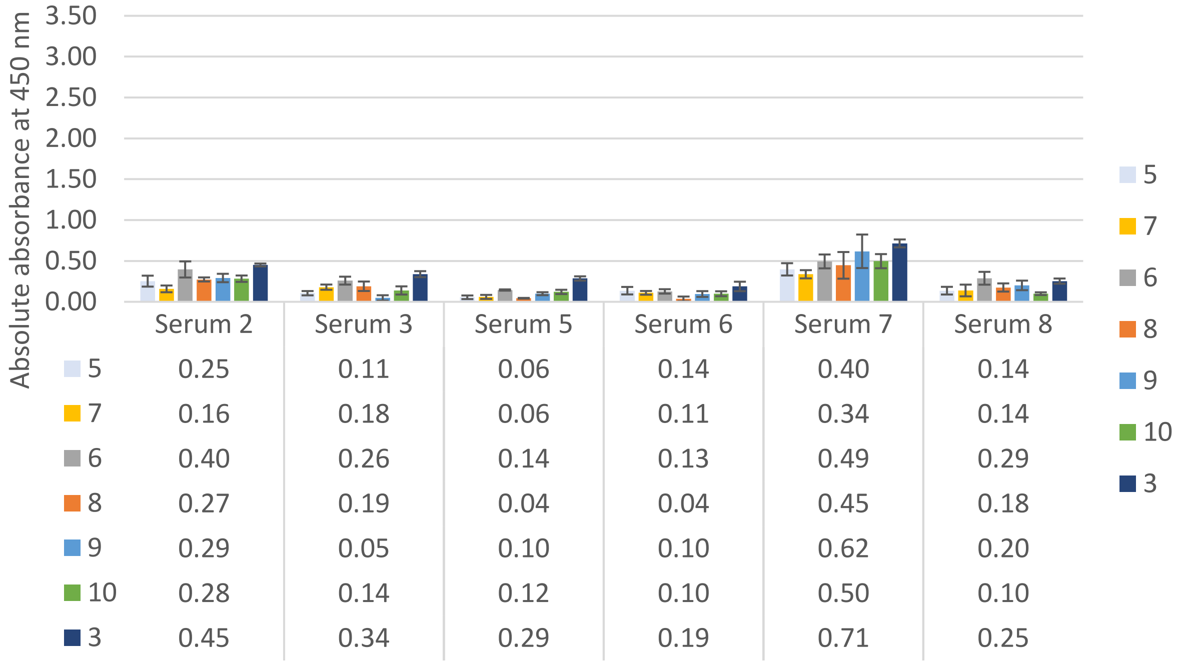

Fig Y. Absolute absorption values obtained in ELISA for the healthy control group.

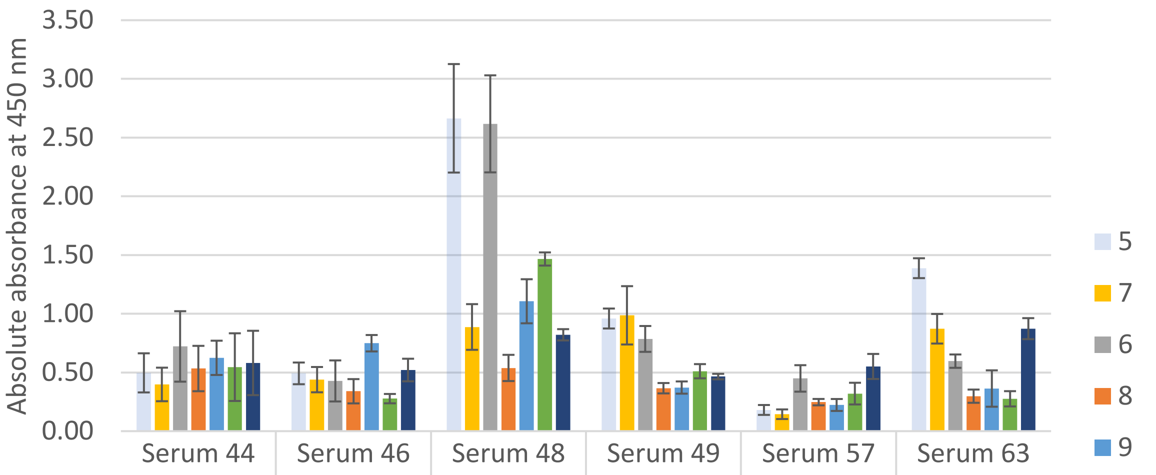

	Serum 44	Serum 46	Serum 48	Serum 49	Serum 57	Serum 63
5	0.50	0.49	2.66	0.96	0.18	1.39
7	0.40	0.44	0.89	0.99	0.14	0.87
6	0.72	0.43	2.62	0.79	0.45	0.60
8	0.53	0.34	0.54	0.37	0.25	0.30
9	0.62	0.75	1.11	0.37	0.22	0.36
10	0.55	0.28	1.47	0.51	0.32	0.28
3	0.58	0.52	0.82	0.46	0.55	0.87

Fig Z. Absolute absorption values obtained in ELISA for the CCP-negative RA patients. The numbers of the sera have been randomly assigned during their collection. The order of the peptides is chosen based on the conformational design: first the all-L reference peptide **5**, then the two mono-D peptides **7** (r9) and **6** (t6), afterwards the double-D motifs **8** (t6,r9) and **9** (s5,s10), finally the tetra-D-sequence **10** (t6,r9,s5,s10) as well as the only bi-disulfide **3** (C1-C14,c5-c10).

Ethical approval

All ELISA experiments utilising blood sera were performed in compliance with the relevant laws and institutional guidelines. Ethical approval was provided by the medical ethics committee of the *Philipps-Universität Marburg*. Collection of human blood sera from both CCP-positive rheumatoid arthritis patients and from the healthy control group has thus been approved by this committee and was executed by medical staff of the *Rheuma-Zentrum Mittelhessen* (Bad Endbach, Germany). Thereby, every participant gave informed consent.

Thiol alkylation and trypsin degradation tetra-thiol sequence 4

Tetra-thiol sequence KcHWEcTCitGRcRLVc **4** was subjected to air oxidation in $(NH_4)_2CO_3$ solution ($c_{peptide}$ ≈ 1 mg/mL). The oxidation proceeded as shown in Fig 3 in the manuscript, tracked by HPLC chromatograms. In the observed presence of the suspected mono-disulfide species, an excess of 2-chloroacetamide was added to the solution. After complete conversion of the mono-disulfide, indicated by complete decline of the corresponding HPLC peak, the solution was lyophilized. For enzymatic degradation, a few micrograms of the crude peptide mixture were dissolved in $(NH_4)_2CO_3$ buffer (50 mM, pH = 8.0) and an excess of trypsin was added. After 5 min at 37 °C, the digestion was stopped by addition of 2 µL of formic acid. The solution was then directly applied to LC-MS measurements.

We searched for fragments with one intact disulfide bridge but broken amide bonds (Fig A'). The first disulfide can be formed between c1-c14 (left path) or c5-c10 (right path), respectively, so that the unreacted thiols are alkylated. These paths only allow specific combinations of fragments upon trypsin digestion (as shown in Fig A', only relevant fragments are depicted). During the LC-MS measurements, only the smaller fragment (M = 390.50 g/mol), that stems from trypsin digestion of the mono-disulfide with c5-c10 (right path), could be detected in relevant amounts (Fig B' to D', intact peptide as well as fragments with and without N-terminal lysine were sought after). Consequently, it can be assumed that c5-c10 forms first in the case of peptide **4**.

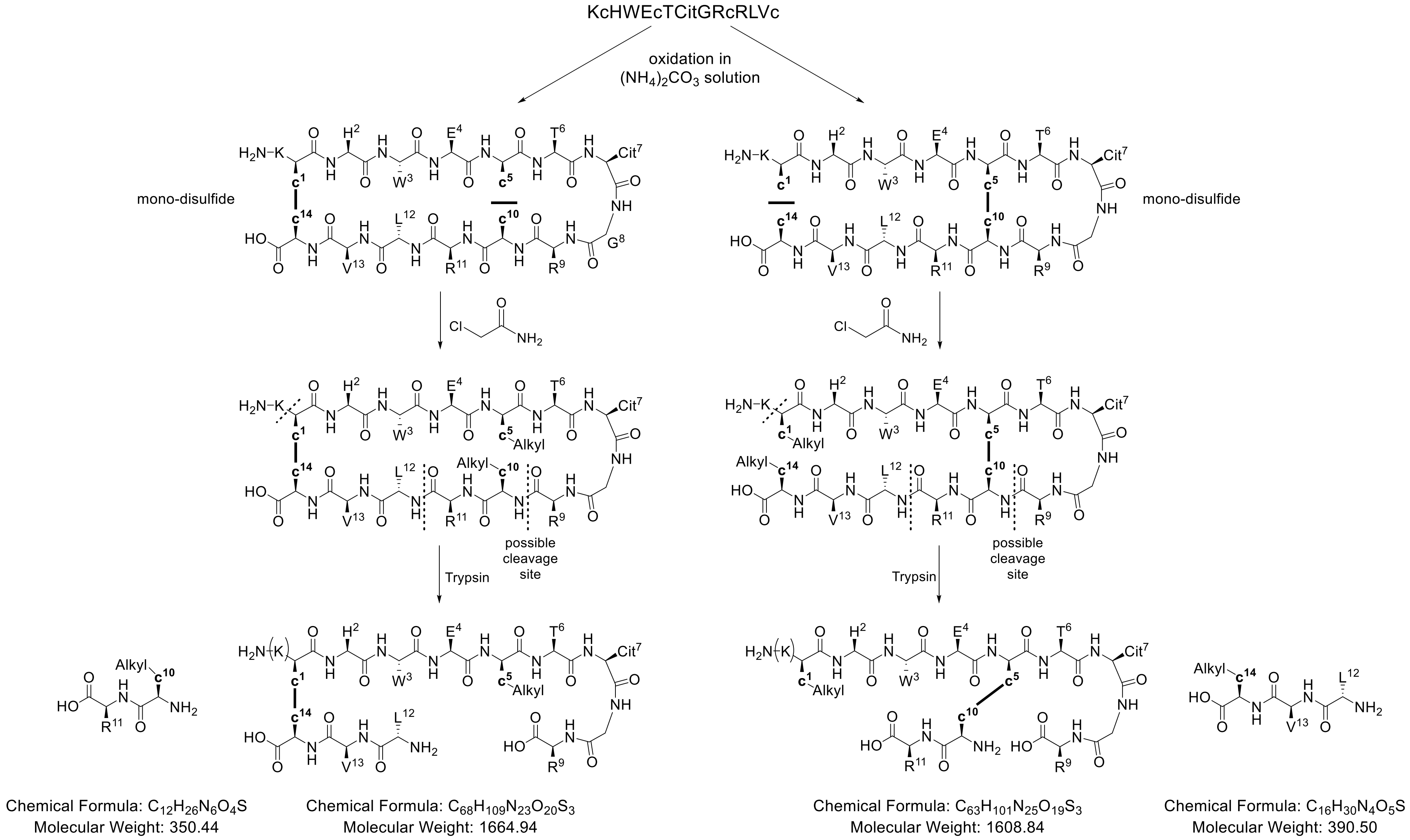

Fig A'. Oxidation and trypsin digestion pathway for tetra-thiol sequence **4**.

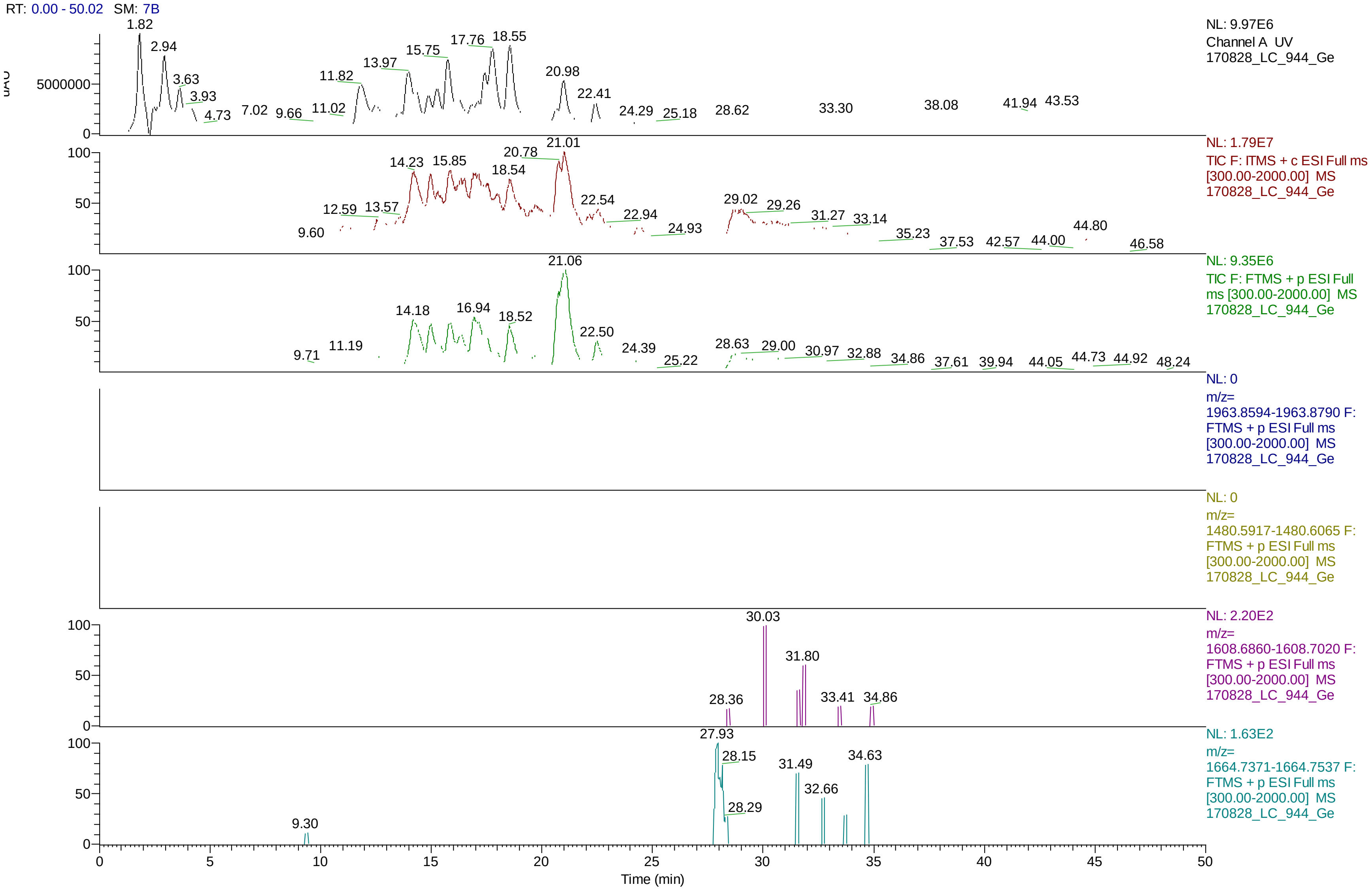

Fig B'. HPLC chromatograms with UV detection (uppermost spectrum) and mass detection.

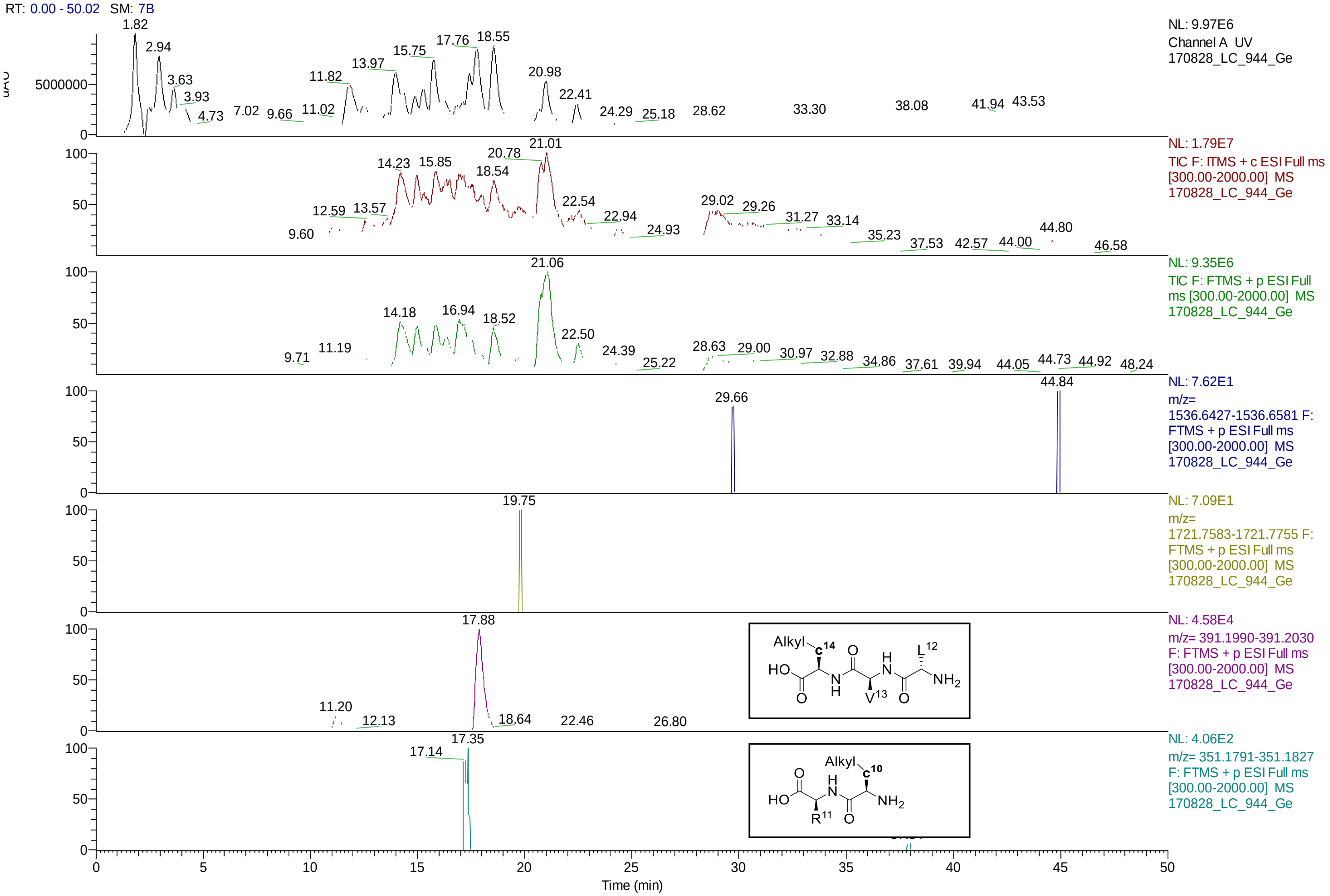

Fig C'. HPLC chromatograms with UV detection (uppermost spectrum) and mass detection.

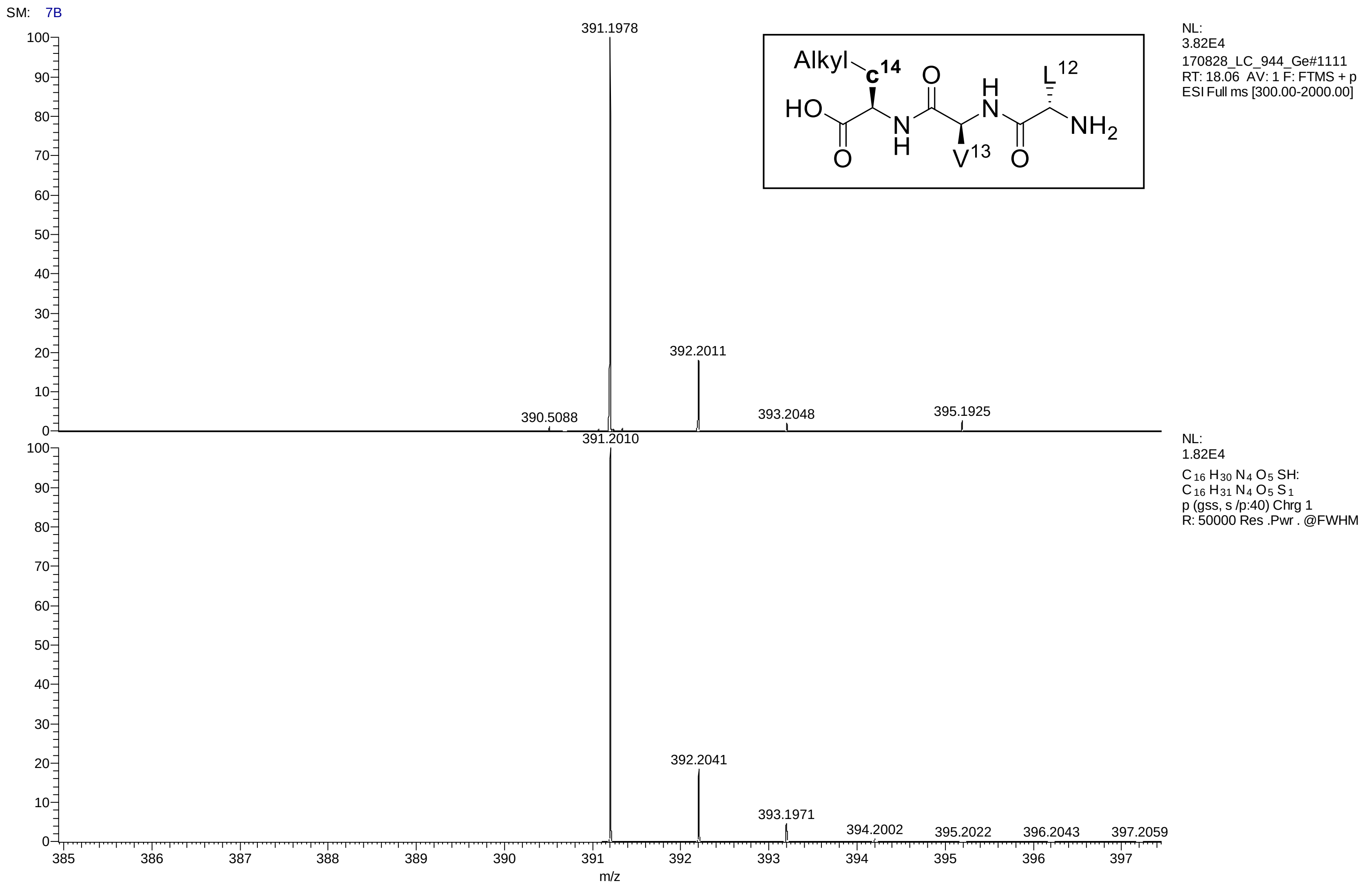

Fig D'. High resolution mass spectrum of the shown fragment, belonging to the mono-disulfide with a disulfide bridge between c5-c10.

Molecular Dynamics Simulation

The modelling of **1d** was carried out as described previously[2,3] using the program package *HyperChem* with the MM+ force field but without explicit water.[4] NOE, hydrogen bond, and side chain torsional restraints were derived from the experimental NMR data and included as target values in the modelling. The final structure shown in Fig 6 of the manuscript was obtained after energy minimisation.[5]

Literature

1. Schrimpf A, Geyer A. Two Opposing D-Amino Acids Give Zigzag Hairpin Epitopes an Additional Kink to Create Antibody-Selective Peptide Antigens. ChemBioChem. 2016;17: 2129–2132. doi:10.1002/cbic.201600479

2. Enck S, Kopp F, Marahiel M a, Geyer A. The entropy balance of nostocyclopeptide macrocyclization analysed by NMR spectroscopy. ChemBioChem. 2008;9: 2597–2601. doi:10.1002/cbic.200800314

3. Haack M, Enck S, Seger H, Geyer A, Beck-Sickinger AG. Pyridone dipeptide backbone scan to elucidate structural properties of a flexible peptide segment. J Am Chem Soc. 2008;130: 8326–8336. doi:10.1021/ja8004495

4. Hypercube, Inc., Gainesville, FL; 2000.

5. DeLano WL. The PyMOL Molecular Graphics System. San Carlos, CA: DeLano Scientific LLC; 2008.
